# A review of heart chamber segmentation for structural and functional analysis using cardiac magnetic resonance imaging

**DOI:** 10.1007/s10334-015-0521-4

**Published:** 2016-01-25

**Authors:** Peng Peng, Karim Lekadir, Ali Gooya, Ling Shao, Steffen E. Petersen, Alejandro F. Frangi

**Affiliations:** Department of Electronic and Electrical Engineering, The University of Sheffield, Sheffield, S1 3JD UK; Universitat Pompeu Fabra, 08002 Barcelona, Spain; Department of Computer Science and Digital Technologies, Northumbria University, Newcastle upon Tyne, NE1 8ST UK; Centre Lead for Advanced Cardiovascular Imaging, William Harvey Research Institute, Queen Mary University of London, London, EC1M 6BQ UK

**Keywords:** Cardiac segmentation, MRI, Clinical assessment

## Abstract

Cardiovascular magnetic resonance (CMR) has become a key imaging modality in clinical cardiology practice due to its unique capabilities for non-invasive imaging of the cardiac chambers and great vessels. A wide range of CMR sequences have been developed to assess various aspects of cardiac structure and function, and significant advances have also been made in terms of imaging quality and acquisition times. A lot of research has been dedicated to the development of global and regional quantitative CMR indices that help the distinction between health and pathology. The goal of this review paper is to discuss the structural and functional CMR indices that have been proposed thus far for clinical assessment of the cardiac chambers. We include indices definitions, the requirements for the calculations, exemplar applications in cardiovascular diseases, and the corresponding normal ranges. Furthermore, we review the most recent state-of-the art techniques for the automatic segmentation of the cardiac boundaries, which are necessary for the calculation of the CMR indices. Finally, we provide a detailed discussion of the existing literature and of the future challenges that need to be addressed to enable a more robust and comprehensive assessment of the cardiac chambers in clinical practice.

## Introduction

Cardiovascular diseases (CVDs) consistently rank among the top major causes of morbidity and mortality. In 2008, 17.3 million people died due to CVDs worldwide, accounting for 30 % of total deaths [1]. Of these cases, about 7.3 million were due to coronary heart disease, and 6.2 million were due to stroke [2]. However, partially due to the aging population , this number keeps increasing. It is predicted that by the year 2030, a population of 23.3 million people will be killed by CVDs all over the world [1, 3]. Consequently, major developments continue to be made in cardiovascular research and practice for improved early diagnosis of cardiac diseases.

In particular, magnetic resonance imaging (MRI), otherwise known as CMR (cardiovascular magnetic resonance), has become a key image modality in clinical practice due to its unique capabilities for non-invasive imaging of the cardiac chambers and great vessels [4]. A wide range of CMR sequences and protocols have been developed to assess various aspects of cardiac function, and significant advances have also been made in terms of imaging quality and acquisition times [5]. Furthermore, a lot of research has been dedicated to the development of global and regional quantitative CMR indices, which help distinguish between pathology and health.

The goal of this review paper is threefold. Firstly, we will review the various functional indices that have been proposed thus far in the literature. These will be presented in detail for each cardiac structure, including their definitions, calculation requirements, the exemplar applications for cardiovascular diseases for each index, and the corresponding normal ranges. Subsequently, and because the calculation of these indices requires delineation of the cardiac boundaries, we will review the most recent state-of-the art techniques for the automatic segmentation of the various cardiac structures. These techniques have the advantage of delineating these boundaries of the heart more rapidly and objectively than clinical experts with manual contouring. We will focus on presenting the more practical properties of these segmentation techniques, such as in relation to their specific roles, and on the imaging materials these techniques need to accomplish the segmentation (e.g. long-axis vs. short-axis images). Finally, we provide a detailed discussion of the existing literature and of the future challenges that need to be addressed to enable a more robust and comprehensive assessment of the cardiac chambers in clinical practice.

In comparison to existing reviews [6–13], our survey is more comprehensive in many aspects, including:

- We review both the segmentation techniques and the structural and functional indices that use the extracted boundaries in their calculations. This will provide a better understanding of the role of cardiac segmentation to the final clinical assessment.
- We also list the quantitative evaluations for the accuracy of both the segmentation and functional analysis to provide the readers with an overview of the level of performance of the existing techniques so far.
- We provide a more comprehensive review of segmentation techniques for all cardiac chambers, including the left ventricle (LV), the right ventricle (RV), the left atrium (LA), and the whole heart.
- We also include the use of long-axis images in cardiac segmentation as they play an important role in clinical use of CMR.
- Finally, we include newly emerged concepts in machine learning-based cardiac image analysis, such as direct estimation of cardiac function [14–17].

This paper is organised as follows. In this introductory section, we will describe the anatomy of the heart, followed by a presentation of the CMR protocols. Subsequently, in section and Table 1, we will present in detail the existing indices of cardiac structure and function, which we will organize per cardiac chamber. In section and Tables 2, 3, 4, 5, and 6, we will then describe the most recent segmentation techniques that can be used to extract automatically the boundaries to be used to compute the functional indices of interest. Finally, we will conclude with a discussion of current cardiac segmentation challenges and future perspectives.

**Table 1:** General recommendations for cardiac functional analysis

	Abbr.	Structure	Calculation methods	Requirement and parameters	Exemplar applications	Normal range
*Left ventricle*						
End-diastolic volume	LVEDV	± Papillary muscles + Outflow tract	Single area-length method Bi-plane area-length method Simpson’s method Direct measurement	2-Chamber LAX view and axis length 2-Chamber, 4-chamber LAX view and axis length Cross-sectional area on each SAX slice and slice thickness A number of contiguous voxels	Dilated cardiomyopathy	M: 156 ± 21 mL, F: 128 ± 21 mL [33] M: 160 ± 29 mL, F: 135 ± 26 mL [34]
End-systolic volume	LVESV	± Papillary muscles + Outflow tract	Single area-length method Bi-plane area-length method Simpson’s method Direct measurement	2-Chamber LAX view and axis length 2-Chamber, 4-chamber LAX view and axis length Cross-sectional area on each SAX slice and slice thickness A number of contiguous voxels	Dilated cardiomyopathy	M: 53 ± 11 mL, F: 42 ± 9.5 mL [33] M: 50 ± 16 mL, F: 42 ± 12 mL [34]
Myocardial mass	LVM	± Papillary muscles and trabecular tissue	(LVV_epi_ − LVV_endo_) × 1.05	LVV_epi_: Left Ventricle Epicardial Volume LVV_endo_: Left Ventricle Endocardial Volume	Hypertension, hypertrophic cardiomyopathy	M: 146 ± 20 g, F: 108 ± 18 g [33] M: 123 ± 21 g, F: 96 ± 27 g [34]
Stoke volume	LVSV		LVEDV − LVESV	End-diastolic and end-systolic volumes	Aortic insufficiency, aortic stenosis	M: 104 ± 14 mL, F: 86 ± 14 mL [33] M: 112 ± 19 mL, F: 91 ± 17 mL [34]
Ejection fraction	LVEF		(LVEDV − LVESV)/LVEDV × 100 %	Stroke volume and end-diastolic volume	Heart failure, hypertrophic cardiomyopathy	M: 67 ± 4.5 %, F: 67 ± 4.6 % [33] M: 69 ± 6.0 %, F: 69 ± 6.0 % [34]
Cardiac output	LVCO		LVCO = LVSV × HR	Stroke volume and heartbeat rate	Hypertension, congestive heart failure	4–8 L/min^a^
Wall thickness	–	− Papillary muscles − Trabecular tissue	Radial method Centreline method	Endocardial and epicardial contours on short-axis image slices, a centre point Endocardial and epicardial contours on short-axis image slices, a centreline and anatomical reference points	Myocardial infarction, hypertension, hypertrophic cardiomyopathy	M: Basel: 7.8 ± 1.1 mm; Mid: 6.3 ± 1.1 mm; Apical: 6.4 ± 1.1 mm, F: Basel: 6.4 ± 0.9 mm; Mid: 5.3 ± 0.9 mm; Apical: 5.9 ± 0.9 mm [35]
Wall thickening	–		(wall thickness_ed_ − wall thickness_es_)/wall thickness_ed_ × 100 %	Average end-systolic wall thickness and average end-diastolic wall thickness		Basal: 73 ± 31 % Mid: 79 ± 26 % Apical: 64 ± 30 % [36]
Strain analysis	LSA	Global coordinates Local coordinates	Lagrangian or Eulerian strain rate Longitudinal, radial, and circumferential strain	Initial location and deformed location Strain tensor	Myocardial infarction, ischemia, and ventricular dyssynchrony	
*Right ventricle*						
End-diastolic volume	RVEDV	± Papillary muscles + Trabecular tissue	Simpson’s method	Cross-sectional area on each slice and slice thickness	Arrhythmogenic right ventricular cardiomyopathy, congenital heart diseases	M: 190 ± 33 mL, F:148 ± 35 mL [34] M:163 ± 25 mL, F: 126 ± 21 mL [37]
End-systolic volume	RVESV	± Papillary muscles + Trabecular tissue	Simpson’s method	Cross-sectional area on each slice and slice thickness	Arrhythmogenic right ventricular cardiomyopathy	M: 78 ± 20 mL, F: 56 ± 18 mL [34] M: 57 ± 15 mL, F: 43 ± 13 mL [37]
Stroke volume	RVSV		RVEDV − RVESV	End-diastolic and end-systolic volume	Pulmonary arterial hypertension	M: 113 ± 19 mL, F: 90 ± 19 mL [34] M: 106 ± 17 mL, F: 83 ± 13 mL [37]
Ejection fraction	RVEF		RVSV/RVEDV × 100 %	Epicardial and endocardial volume	Pulmonary arterial hypertension, congestive heart failure	M: 59 ± 6.0 %, F: 63 ± 5.0 % [34] M: 66 ± 6.0 %, F: 66 ± 6.0 % [37]
Cardiac output	RVCO		RVCO = RVSV × HR	Stroke volume and heartbeat rate	Ventricle failure with cardiomyopathy, pulmonary arterial hypertension	5.25 L/min^b^
*Left atrium*						
Maximum volume	LAV_max_	− Confluence of the pulmonary veins and LA appendage	Single area-length method Bi-plane area-length method Simpson’s method Direct measurement Ellipse method	2-Chamber LAX view and axis length 2-Chamber, 4-chamber LAX view and axis length Cross-sectional area on each SAX slice and slice thickness A number of contiguous voxels Longitudinal diameter, transverse diameter, and antero-posterior diameter	Atrial fibrillation, congestive heart failure, mitral valve disease	M: 103 ± 30 mL, F:89 ± 21 mL [34]
Minimum volume	LAV_min_	− Confluence of the pulmonary veins and LA appendage	Single area-length method Bi-plane area-length method Simpson’s method Direct measurement Ellipse method	2-Chamber LAX view and axis length 2-Chamber, 4-chamber LAX view and axis length Cross-sectional area on each SAX slice and slice thickness A number of contiguous voxels Longitudinal diameter, transverse diameter, and antero-posterior diameter	Atrial fibrillation, congestive heart failure, mitral valve disease	M: 46 ± 14 mL, F: 41 ± 11 mL [34]
Total emptying volume (reservoir)	LAEV	− Confluence of the pulmonary veins and LA appendage	LAV_max_ − LAV_min_	LAV_max_: LA volumes assessed at LV end-systole LAV_min_: LA volumes assessed at late LV end-diastole after LA contraction	Atrial fibrillation, atrial flutter, mitral stenosis, mitral regurgitation, diastolic dysfunction, dilated cardiomyopathy, diabetes mellitus, hypertrophic cardiomyopathy, amyloidosis, and hypertension	
Total emptying fraction (reservoir)	LAEF	− Confluence of the pulmonary veins and LA appendage	(LAV_max_ − LAV_min_)/LAV_max_ × 100 %	LAV_max_: LA volumes assessed at LV end-systole LAV_min_: LA volumes assessed at late LV end-diastole after LA contraction		38 ± 8 % [38]
Passive emptying volume (conduit)	LAPEV	− Confluence of the pulmonary veins and LA appendage	LAV_max_ − LAV_pre A_	LAV_max_: LA volumes assessed at LV end-systole LAV_pre A_: LA volumes assessed at LV diastole just before LA contraction	Atrial fibrillation, atrial flutter, diastolic dysfunction and diabetes mellitus	
Passive emptying fraction (conduit)	LAPEF	− Confluence of the pulmonary veins and LA appendage	(LAV_max_ − LAV_pre A_)/LAV_max_ × 100 %	LAV_max_: LA volumes assessed at LV end-systole LAV_pre A_: LA volumes assessed at LV diastole just before LA contraction		36 ± 11 % [38]
Conduit volume	LACV	− Confluence of the pulmonary veins and LA appendage	LSV − (LAV_max_ − LAV_min_)	LSV: LV stoke volume LAV_max_: LA volumes assessed at LV end-systole LAV_min_: LA volumes assessed at late LV end-diastole after contraction		41 ± 14 mL [39]
Active emptying volume (pump)	LAAEV	− Confluence of the pulmonary veins and LA appendage	LAV_pre A_ − LAV_min_	LAV_pre A_: LA volumes assessed at LV diastole just before LA contraction LAV_min_: LA volumes assessed at late LV end-diastole after LA contraction	Atrial fibrillation, atrial flutter, diastolic dysfunction, dilated cardiomyopathy and diabetes mellitus	
Active emptying fraction (pump)	LAAEF	− Confluence of the pulmonary veins and LA appendage	(LAV_pre A_ − LAV_min_)/LAV_pre A_ × 100 %	LAV_pre A_: LA volumes assessed at LV diastole just before LA contraction LAV_min_: LA volumes assessed at late LV end-diastole after LA contraction		26 ± 3 % [38]
*Right atrium*						
Maximum volume	RAV_max_		3.08 × A_2C_ + 3.36 × A_4C_ − 44.4 Single plane area-length method Bi-plane area-length method	A_2C_ is the area in 2-chamber LAX view and A_4C_ is the area in 4-chamber LAX view 2-Chamber LAX view and axis length 2-Chamber, 4-chamber LAX view and axis length	Chronic heart failure, pulmonary arterial hypertension, tricuspid valve disease, atrial septal defect	M: 109 ± 20 mL, F: 91 ± 20 mL [40]

“+” is include, “−” is exclude, “±” means not specified

*M* male, *F* female

^a^Edwards Lifesciences LLC > Normal Hemodynamic Parameters—Adult 2009

^b^ https://en.wikipedia.org/wiki/Cardiac_output#cite_note-edwards-74

**Table 2:** LV segmentation methods

References	Mode	Dim	Fundamental principles	User interaction	Test cases	Training sets	Materials	Functional analysis performance	Accuracy [distance (mm) and similarity]
Mitchell et al. [119]	Cine	3D	3D AAM	Manual segmentation on training sets	56(18) subjects	Leave 1 out	Multiphase, SAX + LAX	LVV_epi_: 0.97, 0.91, 12.1 (CC&LRC) LVV_endo_: 0.94, 0.88, 8.4 (CC&LRC) LVM: 0.82, 0.80, 17.9 (CC&LRC)	P2S: epi: 2.63 ± 0.76, endo: 2.75 ± 0.86
Paragios [98]	Cine	2D	GVF-based level-sets and contour propagation	–	A few sequences	–	Multiphase, SAX	–	–
Stalidis et al. [95]	Cine	3D + T	Deformable surface modelling + neural network classification	Indicate rough position of cavity and reference samples	3(3) 2D + 1 3D datasets	Guided by user	Multiphase, SAX + LAX	–	–
Santarelli et al. [100]	Cine + P	2D	GVF snake	Draw rough contour of the internal cavity	9 patients (907 images)	–	Multiphase, SAX	–	–
Kaus et al. [104]	Cine	3D	Prior (coupled meshes + PDM) + deformable model	Manual segmentation on training sets	121 subjects	Leave 1 out	ED + ES, SAX	–	epi: 2.92 ± 1.38 (ES), 2.62 ± 0.75 (ED) endo: 2.76 ± 1.02 (ES), 2.28 ± 0.93 (ED)
Yeh et al. [132]	Cine	2D	DP-based border detection	Place region of interest which includes the whole LV	1(0) subjects	–	Multiphase, SAX	–	–
Gotardo et al. [106]	Cine	2D + T	Fourier shape constraints + deformable model tracking	Specify 4 points on the desired boundary in one image	33(33) subjects	*Leave 1 out for classifier*	Multiphase, SAX	–	–
Jolly [92]	Cine	2D	LV localisation + EM based classification + active contours	Crop the image to limit the localisation search space	29 patients (482 images)	–	ED + ES, SAX	–	–
Pednekar et al. [94]	Cine	2D + T	Motion-map and EM guided localisation + DP-based walls extraction	–	14 subjects	–	Multiphase, SAX	LVESV error: −10.90 mL, LVEDV error: −0.17 mL LVEF error: 7.21 %	–
van Assen et al. [120]	Cine	3D	3D ASM + fuzzy inference	Manual segmentation on the basal and apical slices	15(0) subjects + 5(5) patients	Pre-constructed atlas	–, SAX/LAX	LVV_epi_: 219.3 ± 41.3 mL (SAX), 243.0 ± 35.0 mL (RAD), 229.1 ± 41.6 mL (MV) LVV_endo_: 122.0 ± 27.3 mL (SAX), 132.5 ± 18.5 mL (RAD), 127.5 ± 28.1 mL (MV)	epi: 2.23 ± 0.46 (SAX), 2.83 ± 0.78 (RAD), 2.29 ± 0.53 (MV) endo: 1.97 ± 0.54 (SAX), 2.24 ± 0.54 (RAD), 2.02 ± 0.93 (MV)
Lynch et al. [105]	Cine	2D	Region-based coupled level-set	Manual insertion of a seed point	4 slices	–	–, SAX	–	P2C: endo: 0.477 ± 0.683, epi: 1.149 ± 1.157
Lekadir et al. [121]	Cine	3D	3D ASM + outlier correction	Manual segmentation on training sets	36 subjects	Leave 1 out	–, SAX	–	epi: 1.11 ± 0.46, endo: 0.78 ± 0.21
Andreopoulos and Tsotsos [122]	Cine	3D + T	3D AAM + hierarchical 2D ASM with temporal constraints	Indicate a few endocardial and myocardial regions	33 subjects (7980 slices)	Threefold cross validation	Multiphase, SAX	LVV_epi_: 0.97, 0.98, 2.7 (CC&LRC) LVV_endo_: 0.95, 0.92, 4.6 (CC&LRC)	–
Chen et al. [110]	DENSE	2D	Optimal boundary initialisation + deformable model	Crop the input image to put LV centroids in the centre	5(0) healthy	–	Multiphase, SAX	–	–
Codella et al. [85]	Cine	2D	Region growth + seeds propagation	Choose the mid-ventricular slice	38(20) subjects	–	Multiphase, SAX	LVESV error: −1.9 ± 6.1 mL LVEDV error: 0.8 ± 5.1 mL LVV: 0.99, 0.98, −2.9 (CC&LRC) LVEF error: 3 ± 7.5 % LVEF: 0.95, 1.01, −1.9 (CC&LRC)	–
Folkesson et al. [96]	LE	2D	Geodesic active region + statistical KNN classifier	Manual segmentation on training sets	4 patients (30 slices)	7 patients (57 slices)	–, SAX	–	1.44 ± 0.54 (P2C); 0.79 ± 0.07 (DC)
Huang and Metaxas [111]	TA	2D	Deformable shape and appearance model	Specify the centroid and the radius by 2 clicks	–	–	–	–	–
Lynch et al. [108]	Cine	3D + T	Level-set + temporal prior + EM optimised fitting	–	6 subjects	A set of boundaries	Multiphase, SAX	–	endo: 1.25 ± 1.33 (P2C)
Sun et al. [109]	Cine	3D + T	Level-set + recursive estimation using temporal learning	Manual segmentation on training sets	26 patients (234 cycles, 4680 slices)	5 patients (42 cycles, 840 slices)	Multiphase, SAX		endo: 0.93 (DC)
van Assen et al. [123]	Cine	3D	3D ASM + fuzzy inference	Place landmark at the posterior junction of RV and LV in a mid-ventricular slice	15(0) subjects	53 subjects	ED, SAX/LAX	LVV_epi_: 0.99, 0.94, 27.8 (CC&LRC) LVV_endo_: 0.99, 0.85, 6.62 (CC&LRC)	P2P: epi: 1.27–1.85, endo: 1.34–2.05
Kermani et al. [115]	Cine	3D	3D active mesh model	–	Synthetic + 6(1) real sequences	–	Multiphase, SAX	LV volume error: 3.77 ± 1.67 % LVM error: 5.26 ± 1.71 %	–
Kurkure et al. [90]	Cine	2D	Fuzzy connectedness based region growth	Manual segmentation on 3 ED mid-ventricle slices per subject	20 (15) subjects	–	Multiphase, SAX + LAX	LVESV error: 8.82 ± 11.91/−3.80 ± 6.99 mL LVEDV error: −16.02 ± 19.69/−2.96 ± 7.57 mL LVEF error: 1.57 ± 5.17 %/1.83 ± 5.70 %	endo: 0.86 ± 0.12 (DC)
Spottiswoode et al. [112]	DENSE	2D + T	Motion trajectory guided contour propagation	Draw the initial myocardial contours on any frame	6(0) subjects	–	Multiphase, SAX/LAX	–	Radial C2C: epi: 1.01 ± 0.23, endo: 1.29 ± 0.34
Suinesiaputra et al. [124]	Cine	2D	ICA statistical model based detection and classification	Manual segmentation on training sets	45(45) subjects	44(0) volumes	ED + ES, SAX	–	–
Constantinides et al. [101]	Cine	2D	GVF based deformable model + fuzzy k-means papillary muscle detection	Place a point at the centre of LV and a point at the upper intersection of LV and RV	15(12) subjects	15(12) subjects	Multiphase, SAX	LEF: 0.97, 1.00, 1.60 (CC&LRC) LVM: 0.88, 0.80, 31.51 (CC&LRC)	APD: epi: 2.35 ± 0.57, endo: 2.04 ± 0.47 DC: epi: 0.92, endo: 0.89
Chen et al. [114]	TA	3D + T	Deformable model based motion tracking	–	17(11) subjects	–	Multiphase, SAX + LAX	–	–
Cousty et al. [91]	Cine	3D + T	Morphological region growth + watershed cuts	Specify a single point located at the centre of cavity	18(18) subjects	–	Multiphase, SAX	–	P2S: epi:1.55 ± 0.23, endo: 1.42 ± 0.36
Lee et al. [84]	Cine	2D	Region growth with iterative thresholding + active contours	Choose mid-ventricular slice	38 patients (339 images)	–	Multiphase, SAX	LVV_epi_: 0.98 (CC), 2.0 ± 13.0 mL (error) LVV_endo_: 0.99 (CC), 2.9 ± 6.2 mL (error) LVM: 0.90, 0.93, 10.37 (CC&LRC), −0.9 ± 16.5 g (error)	–
Schaerer et al. [107]	Cine	2D + T	Deformable elastic template + temporal constraints	Specify a point at the centre of cavity in the ED frame	15(15) subjects	–	Multiphase, SAX	LV volume error: −12–57 % LVEF error: 1–6 % LVM error: 4–35 %	APD: epi: 3.14 ± 0.33, endo: 2.97 ± 0.38 DC: epi: 0.92 ± 0.02, endo: 0.87 ± 0.04
Zhu et al. [130]	Cine	3D + T	Propagation based subject-specific dynamic model	Manual segmentation on first frame can be required	22(0) subjects	Leave 1 out	Multiphase, SAX	LVV error: −2.3 to 0.5 mL	MAD: epi:1.27 ± 0.18, endo: 0.69 ± 0.13 HD: epi:1.72 ± 0.15, endo: 1.47 ± 0.16
Cordero-Grande et al. [118]	Cine	3D + T	Markov random field based deformable model	–	43(43) subjects	–	Multiphase, SAX	LVESV: −7.19, 1.05 (LRC); error: −3.3 ± 7.2 mL LVEDV: −1.59, 0.99 (LRC); error: −3.6 ± 8.2 mL LVEF: −2.23, 1.07 (LRC); error: 1.5 ± 3.3 % LVM: 2.22, 1.06 (LRC); error: 8.2 ± 11.6 g	S2S: epi: 1.22 ± 0.17, endo: 1.37 ± 0.20
Huang et al. [86]	Cine	2D	Thresholding + edge detection + radial region growth	Choose mid slice and manual correction can be required	45(36) subjects	–	Multiphase, SAX	–	APD: epi: 2.22 ± 0.43, endo: 2.16 ± 0.46 DC: epi: 0.93 ± 0.02, endo: 0.89 ± 0.04
Lekadir et al. [126]	Cine	3D + T	PDM + local spatial–temporal descriptor	Place 4 landmarks	50 subjects	Cross validation	Multiphase, SAX + LAX	–	1.46 ± 0.35
Brien et al. [127]	Cine	3D + T	ASM + global contour optimisation	Manual segmentation on training sets	*n* subjects	33-*n* subjects	Multiphase, SAX + LAX	LVV_epi_: 0.97–0.99 (CC) LVV_endo_: 0.88–0.95 (CC)	–
Ammar et al. [88]	Cine/LE/P	2D	Thresholding + level-set	–	18(18) subjects	–	Multiphase, SAX	–	–
Ayed et al. [133]	Cine	2D	Subject-specific model + max-flow optimisation	Manual segmentation on first frame	20 subjects (2280 slices)	–	Multiphase, SAX	LVV_endo_: 0.99 (CC) Myocardial volume: 0.81 (CC)	DC: endo: 0.92 ± 0.03, myocardium: 0.82 ± 0.06
Khalifa et al. [116]	Cine	3D	Level-set based geometric deformable model with prior	Manual segmentation on training sets	26(26) subjects	1/3 of total	Multiphase, SAX	–	APD: epi: 0.87 ± 0.52, endo: 1.21 ± 1.29 DC: epi: 0.96 ± 0.02, endo: 0.91 ± 0.07
Ringenberg et al. [102]	Cine + LE	2D	Thresholding and Canny edge detection based ROI extraction + GVF snake	–	5(5) subjects	–	Multiphase, SAX	–	P2C: Cine: epi: 1.45 ± 0.65, endo: 1.25 ± 0.39; LE: epi: 1.95 ± 0.85, endo: 1.73 ± 0.69
Eslami et al. [135]	Cine	3D + T	Retrieval closet subject with guided random walks	Provide myocardial and background seeds on ED frame	104(73) subjects	–	Multiphase, SAX	LVESV: 0.98, 0.96, 2.10 (CC&LRC) LVEDV: 0.98, 0.92, 1.58 (CC&LRC) LVEF: 0.96, 0.99, 1.61 (CC&LRC) LVM: 0.95, 1.04, 3.40 (CC&LRC)	P2C: epi: 1.48 ± 0.44, endo: 1.54 ± 0.31 DC(%): endo: 83.25 ± 3.05 (ED), 84.69 ± 4.17 (ES); epi: 80.15 ± 3.89 (ED), 79.65 ± 5.04 (ES)
Hu et al. [93]	Cine	2D	GMM (EM) + region restricted dynamic programming	–	45(36) subjects	–	Multiphase, SAX	LVEF: 0.94, 1.01, 2.76 (CC&LRC) LVM: 0.82, 0.90, 9.9 (CC&LRC)	APD: epi: 2.21 ± 0.45, endo: 2.24 ± 0.40 DC: epi:0.94 ± 0.02, endo: 0.89 ± 0.03
Lu et al. [87]	Cine	2D	Optimal thresholding + FFT + multiple seeds region growth	Choose mid-slice and manual correction can be required	133(96) subjects	–	Multiphase, SAX	LVEDV: 0.98 (CC), LVESV: 0.98 (CC) LVEF: 0.90 (CC), LVM: 0.88 (CC)	APD: epi: 0.92, endo: 2.08 DC: epi: 0.94, endo: 0.90
Nambakahsh et al. [134]	Cine	3D	Convex relaxed + distribution matching with priors	Specify a single point on target region (cavity or myocardium)	20 subjects (400 volumes)	Leave 1 in	Multiphase, SAX	LVV_epi_: 0.91 (CC) LVV_endo_: 0.88 (CC)	DC: epi: 0.70 ± 0.01, endo: 0.80 ± 0,02
Roohi and Zoroofi [128]	Cine	3D + T	Kernel PCA based 3D ASM	Manual segmentation on training sets	33 subjects (7980 slices)	Leave 1 out	Multiphase, SAX	LVV_epi_: 0.99, 1.92 (LRC) LVV_endo_: 1.00, 1.61 (LRC)	–
Wei et al. [117]	Cine + LE	3D	Propagate contours prior from cine to LE + deformable model	Exclude the most basal and apical slices and selects one 4-chamber and one 2-chamber LAX slices from LE images	12 patients, 4 simulated phantom data	–	One phase, SAX + LAX	–	epi: 0.67 ± 0.41, endo: 0.73 ± 0.49 DC(%): 98.05 ± 0.07
Woo et al. [99]	Cine	2D	Coupled level-set + dual shape constraint	Choose centre of endocardium and its boundary by 2 clicks on mid-slice at ED	15 subjects	–	Multiphase, SAX	LVESV: 68 ± 49 mL (Grd 69 ± 45 mL) LVEDV: 139 ± 44 mL (Grd 139 ± 41 mL) LVEF(%): 54 ± 16 (Grd 55 ± 19)	DC: 0.89 ± 0.03
Wu et al. [103]	Cine	2D	GVC based parametric active contour	–	126(0) + 45(45) images	–	Multiphase, SAX	–	MAD: epi: 5.18 pixels, endo: 5.06 pixels
Afshin et al. [14]	Cine	2D	Image feature + LDA + linear SVM classification	Specify initial segmentation and anatomical landmarks on the first SAX slice	58(37) subjects	Threefold cross validation	Multiphase, SAX	Classification accuracy: 86.09 %	–
Alba et al. [131]	Cine/LE	3D	Intensity based graph-cuts + inter-slice and shape constraint	–	15 cine + 20 LE patients	–	Multiphase, SAX	–	P2P: Cine: epi: 2.58 ± 0.39, endo: 2.76 ± 0.53; LE: epi: 2.38 ± 0.53, endo: 1.83 ± 0.50 DC: Cine: 0.92 ± 0.04, LE: 0.81 ± 0.05
Auger et al. [113]	DENSE	3D	Displacement based contour propagation + model fitting	Specify guide points on myocardial borders on 3 SAX slices (apical, mid, and basal)	4(0) subjects	–	Multiphase, SAX	–	DC: 0.92
Qin et al. [129]	Cine	2D	Feature competition + sparse model + incremental learning	Manual segmentation on first frame	33 subjects (mid slices)	Leave 1 out	Multiphase, SAX	–	P2C: epi: 1.44 ± 0.36, endo: 1.75 ± 0.50 DC: epi: 0.95 ± 0.01, endo: 0.90 ± 0.03
Queiros et al. [89]	Cine	3D + T	B-spline explicit active surface + sequential thresholding + EM	Choose basal and apical slices by 2 clicks	45(36) subjects	–	Multiphase, SAX	LVEDV: 0.985, 0.99, −1.04 (CC&LRC) LVESV: 0.988, 1.026, −6.903 (CC&LRC) LVM: 0.951, 1.04, 0.69 (CC&LRC) LVEF: 0.976, 1.10, −1.63 (CC&LRC)	APD: epi: 1.80 ± 0.41, endo: 1.76 ± 0.45 DC: epi: 0.94 ± 0.02, endo: 0.90 ± 0.05
Bai et al. [97]	Cine	3D	Multi-atlas + augmented feature + SVM classification	Place 5 landmarks on ED frames in the target and atlas	83 subjects	Leave 1 out	Multiphase, SAX	LVESV error: 9.3 ± 9.9 mL LVEDV error: 8.9 ± 8.2 mL LVM error: 11.9 ± 12.4 g	DC: 0.807

*TA* tagged CMR, *LE* LGE CMR, *P* perfusion CMR, *DC* dice similarity coefficient (ideally 1), *CC* correlation coefficient (ideally 1), *LRC* linear regression coefficients (*y* = *ax* + *b*, ideally *a* = 1, *b* = 0); “+T” temporal information is incorporated; *P2P*, *P2C*, *P2S*, *S2S*, *APD*, *MAD*, and *HD* are point-to-point, point-to-curve, point-to-surface, surface-to-surface, average perpendicular distance, mean absolute Distance, and Hausdorff distance, respectively; 45(36) means 36 out of 45 subjects are abnormal or unhealthy

**Table 3:** RV segmentation methods

References	Mode	Dim	Fundamental principles	User interaction	Test cases	Training sets	Materials	CC and LRC	HD (mm)	DC
Maier et al. [136]	Cine	3D + T	Region-growing (watershed) + graph-cut	Specify the midline of RV wall in ED slices or 2 points on ED basal slice for registration	16(16) subjects	16(16) subjects if atlas is in use	Multiphase, SAX	RVESV: 0.96, 1.06, 6.73 RVEDV: 0.99, 1.06, 1.02 RVEF: 0.86, 0.07, −0.06	endo: 14.75 ± 0.40 (ES), 9.21 ± 0.29 (ED)	endo: 0.69 ± 0.02 (ES), 0.84 ± 0.01(ED)
Ou et al. [142]	Cine	3D	Atlas registration based propagation + label fusion	–	16(16) subjects	16(16) subjects	ED + ES, SAX	–	epi: 21.91 ± 18.92 (ES), 19.21 ± 18.50 (ED) endo: 20.44 ± 17.80 (ES), 18.77 ± 18.29 (ED)	epi: 0.60 ± 0.30 (ES), 0.69 ± 0.28 (ED) endo: 0.53 ± 0.32 (ES), 0.65 ± 0.30 (ED)
Wang et al. [117]	Cine	3D + T	X–Y direction spatial morphological patterns + Z and temporal refinement	–	16(16) subjects	–	Multiphase, SAX	RVESV: 0.80, 1.56, 2.30 RVEDV: 0.87, 1.37, 5.14 RVEF: 0.29, 0.48, 0.19	epi: 27.58 ± 24.82 (ES), 21.45 ± 25.14 (ED) endo: 27.99 ± 24.97 (ES), 22.89 ± 25.01 (ED)	epi: 0.55 ± 0.36 (ES), 0.70 ± 0.34 (ED) endo: 0.50 ± 0.34 (ES), 0.63 ± 0.32 (ED)
Zuluaga et al. [143]	Cine	2D	Atlas based coarse-to-fine segmentation + label fusion	–	16(16) subjects	16(16) subjects	ED + ES, SAX	RVESV: 0.97, –, – RVEDV: 0.96, –, –	epi: 11.81 ± 9.46 (ES), 10.23 ± 7.22 (ED) endo: 11.41 ± 10.49 (ES), 9.77 ± 7.88 (ED)	epi: 0.77 ± 0.23 (ES), 0.86 ± 0.13 (ED) endo: 0.72 ± 0.27 (ES), 0.83 ± 0.17 (ED)
Bai et al. [144]	Cine	3D	Multi-atlas registration + label fusion	Specify a few landmarks on ED slices for registration	16(16) subjects	16(16) subjects	ED + ES, SAX	RVESV: 0.98, 0.67, 12.13 RVEDV: 0.99, 0.87, 17.86 RVEF: 0.92, 0.57, 0.29 RV mass: 0.91,0.82, 1.35	epi: 11.72 ± 5.44 (ES), 7.93 ± 3.72 (ED) endo: 11.16 ± 5.53 (ES), 7.70 ± 3.74 (ED)	epi: 0.77 ± 0.17 (ES), 0.88 ± 0.08 (ED) endo: 0.69 ± 0.25 (ES), 0.86 ± 0.11 (ED)
Nambakhsh et al. [141]	Cine	3D	Prior distribution matching + convex relaxation	Specify the centroid of LV and a small closed region inside RV cavity in the middle slice	32(32) subjects	Leave 1 in	ED + ES, SAX	RVESV: 0.79, 1.05, 52.04 RVEDV: 0.81, 1.02, 36.48 RVEF: 0.28, 0.38, 0.10	endo: 23.19 ± 9.71 (ES), 17.76 ± 7.73 (ED)	endo: 0.48 ± 0.25 (ES), 0.67 ± 0.19 (ED)
Grosgeorge et al. [145]	Cine	2D	Distance map-based SSM + registration + graph cut	Place 2 anatomical landmarks on the ventricular septum	16(16) subjects	16(16) subjects	ED + ES, SAX	–	–	endo: 0.70 ± 0.22 (ES), 0.83 ± 0.15 (ED)
Mahapatra [140]	Cine	2D/3D	Super-pixel or super-voxel classification by random forest	–	32 datasets	Leave 1 out	Multiphase, SAX	–	endo: 6.7	endo: 0.93
Oghli et al. [146]	Cine	2D	Robust PCA shape based deformable model	Manual segmentation on training sets	30(30) slices	30 binary shapes	ED + ES, SAX	–	–	–
Ringenberg et al. [138]	Cine	2D	PCA window constraints + accumulator thresholding	Manual segmentation on training sets	32(32) subjects	16(16) subjects	ED + ES, SAX	RVESV: 0.95, 1.02, 10.16 RVEDV: 0.98, 1.10, −6.64 RVEF: 0.78, 0.83, 0.02 RV mass: 0.97, 1.10, −2.77	epi: 11.52 ± 7.70 (ES), 8.02 ± 5.96 (ED) endo: 10.71 ± 7.69 (ES), 7.69 ± 6.03 (ED)	epi: 0.82 ± 0.13 (ES), 0.90 ± 0.08 (ED) endo: 0.77 ± 0.18 (ES), 0.88 ± 0.11 (ED)
Punithakumar et al. [139]	Cine	2D + T	Moving mesh propagation by point-to-point correspondence	Manual segmentation on a single initial frame	48(48) +23(23) subjects	–	Multiphase, SAX	–	epi: 8.08 ± 3.80 endo: 7.72 ± 3.97	epi: 0.87 ± 0.08 endo: 0.83 ± 0.13

*DC* dice similarity coefficient (ideally 1), *CC* correlation coefficient (ideally 1), *LRC* linear regression coefficients (*y* = *ax* + *b*, ideally *a* = 1, *b* = 0); “+T” temporal information is incorporated; *HD* error in Hausdorff distance; 30(30) means 30 of 30 subjects are abnormal or unhealthy

**Table 4:** Bi-ventricle segmentation methods

References	Mode	Dim	Fundamental principles	User interaction	Test cases	Training sets	Materials	CC and LRC	LV_endo_/LV_epi_/RV distance (mm)
Mitchell et al. [158]	Cine	2D	ASM + AAM	Manual segmentation on training sets	60(27) mid-ventricle slices	102(33) mid-ventricle slices	ED, SAX	LV_epi_: 0.96, 0.90, 0.41 LV_endo_: 0.96, 1.04, −0.55 RV: 0.90, 0.97, −0.22	P2P(signed): 0.22 ± 1.90/−0.01 ± 1.92/−0.32 ± 2.80
Ordas et al. [157]	Cine	2D	ASM + invariant optimal features	Manual segmentation on training sets	74(61) subjects	21(13) subjects	Multiphase, SAX	–	P2C: 1.80 ± 1.74/1.52 ± 2.01/1.20 ± 1.74
Sermesant et al. [147]	Cine	3D + T	Deformable biomechanical mesh registration + tracking	Choose reasonable mesh size	2 sequences	–	Multiphase, SAX + LAX	–	–
Lorenzo-Valdes et al. [155]	Cine	3D + T	4D probabilistic atlas + MRF + EM algorithm	Manual segmentation on training sets	14(0) + 10(10) subjects	Leave 1 out	Multiphase, SAX	LVV_epi_: 0.92, 1.18, 7.0 LVV_endo_: 0.96, 0.92, −3.42 RV volume: 0.92, 0.90, 15	P2C: 2.21 ± 2.22/2.99 ± 2.65/2.89 ± 2.56
Rougon et al. [148]	Cine + TA	2D	Dense motion estimation + non-rigid propagation from ED	–	12 subjects	–	Multiphase, SAX + LAX	–	–
Hautvast et al. [149]	Cine	2D	Automatic contour propagation from ED slices to ES slices	Segment an ED frame as initialisation	69(69) SAX slices + 38(38) LAX slices	–	Multiphase, SAX/LAX	LVESV: SAX: 0.98, 1.03, −2.08; LAX: 0.93, 0.92,13.72 LVSV: SAX:0.71, 0.78, 9.74; LAX: 0.68, 0.61, 11.19 LVEF: SAX: 0.78, 0.99, 2.08; LAX: 0.76, 0.59, 12.43	SAX (ES): 2.23 ± 1.10/1.84 ± 1.04/2.02 ± 1.21 LAX (ES): 1.82 ± 0.61/0.92 ± 0.42/–
Cocosco et al. [150]	Cine	3D + T	Binary voxel classification + thresholding + region-growing	Choose basal slices	32(32) subjects	–	Multiphase, SAX	LVV_endo_: 0.97, 0.94, 15.7 RV volume: 0.97, 1.11, 17.9 LVEF: 0.94, 0.98, −7.58 RVEF: 0.71, 0.60, 14.3	–
Zhang et al. [159]	Cine	3D + T	ASM + AAM	Fitting the mean shape prior to the first frame as initialisation	25(0) + 25(25) subjects	Fivefold cross-validation	Multiphase, SAX + LAX	LVV_endo_: 0.98, 0.97, 7.1 RV volume: 0.96, 0.95, 3.0 LVM: 0.76, 0.88, 13.9	P2S (normal): 1.67 ± 0.30/1.81 ± 0.40/2.13 ± 0.39 P2S (TOF): 1.71 ± 0.45/1.97 ± 0.58/2.92 ± 0.73
Grosgeorge et al. [151]	Cine	2D	Region-based level-set	–	59(59) subjects	–	ED + ES, SAX	–	P2C (ED):2.33–3.52/–/2.27–3.28 P2C (ES): 2.27–5.00/–/1.85–3.50 DC (ED): 0.67–0.82/–/0.46–0.80 DC (ES): 0.46–0.70/–/0.25–0.59
Mahapatra [153]	Cine	2D	Single shape prior + graph-cut	Identify myocardium, LV and RV in the first frame	30(30) subjects	–	Multiphase, SAX	–	HD: 1.8 ± 0.4/1.9 ± 0.3/2.0 ± 0.3 DC (%): 91.7 ± 1.1/91.6 ± 0.9/92.2 ± 1.2
Wang et al. [154]	Cine	3D	Context-specific reinforcement learning	Place points on the correct contour during segmentation	60(0) + 21(21) subjects	15 subjects when segmenting RV	Multiphase, SAX	–	C2C (ED): 0.91 ± 0.18 (healthy LV_endo_)/1.73 ± 0.64 (healthy RV)/1.15 ± 0.25 (HCM LV) C2C (ES): 1.01 ± 0.20 (healthy LV_endo_)/2.32 ± 0.96 (healthy RV)/1.17 ± 0.24 (HCM LV)
Bai et al. [156]	Cine	3D	Multi-atlas registration + patch based probabilistic label fusion	Manual segmentation on training sets	28(0) subjects	Leave 1 out	Multiphase, SAX	–	Average: 1.26/1.49/1.68 Maximum: 7.27/9.35/12.23 DC: 0.915/0.824/0.886
Wang et al. [15]	Cine	2D	Prior probability model + direct area estimation	Place 2 landmarks on each slice in first frame	56 subjects (3360 slices)	Leave 1 out	Multiphase, SAX	LVV_endo_: 0.985, –, – RV volume: 0.957, –, – LVEF: 0.966, –, – RVEF: 0.807, –, –	–
Zhen et al. [16]	Cine	2D	Direct estimation by multiscale deep networks and regression forest prediction	Manual segmentation on training sets	100 subjects (6000 slices)	Unsupervised feature learning: 47 subjects (2820 slices) Supervised learning: Leave 1 out validation in 100 subjects	Multiphase, SAX	LVV_endo_: 0.921, –, – RV volume: 0.908, –, –	–
Alba et al. [160]	Cine	3D	PDM based feature searching + model fitting in various pathologies	Specify a few landmarks	20 normal as reference + 40(40) subjects	Leave 1 out	ED, SAX	–	P2S: pulmonary hypertension: 2.60 ± 0.34; hypertrophic cardiomyopathy: 2.57 ± 0.46

*TA* tagged CMR, “+T” temporal information is incorporated, *DC* dice similarity coefficient (ideally 1), *CC* correlation coefficient (ideally 1), *LRC* linear regression coefficient (*y* = *ax* + *b*, ideally *a* = 1, *b* = 0); *P2P*, *P2C*, *P2S*, *C2C,* and *HD* are point-to-point, point-to-curve, point-to-surface, curve-to-curve, and Hausdorff distance, respectively; 60(27) means 27 out of 60 subjects are abnormal or unhealthy

**Table 5:** LA segmentation methods

References	Protocol	Dimension	Fundamental principles	User interaction	Test cases	Training sets	Accuracy
John and Rahn [161]	F	2D	Thresholding + subdivision (narrow cuts) + region merging	Final segmentation positive and negative marking	20 subjects	–	–
Karim et al. [163]	LE	2D	3D probabilistic atlas construction + MRF based energy function minimisation within Voronoi framework	Choose 3 or 4 landmarks on each training image	10(10) volumes	20(20) volumes	Mean slice overlap: 0.90
Kutra et al. [164]	Cine/LE	3D	Multi-model based fitting + SVM based optimal model selection	Manual segmentation on training sets	59(47 %) subjects	Leave 1 out validation	P2S: Normal: 0.87 mm; CLT: 0.81 mm; RMPV: 0.79 mm
Zhu et al. [162]	LE	2D	Local seed region searching + region growth with prior	Manual segmentation on training sets	64(64) volumes	16 volumes	DC: 0.79 ± 0.05, Volume overlap: 0.65 ± 0.07, HD: 14.40 ± 3.65 mm, S2S: 2.79 ± 2.84 mm

*F* flow CMR, *LE* LGE CMR, *DC* dice similarity coefficient (ideally 1), *P2S* point-to-surface, *HD* Hausdorff distance; 64(64) means 64 out of 64 subjects are abnormal or unhealthy

**Table 6:** Whole heart segmentation methods

References	Mode	Dim	Fundamental principles	User interaction	Test cases	Training sets	Materials	S2S (mm)						
								LV_epi_	LV_endo_	RV_epi_	RV_endo_	LA	RA	Whole mesh
Makowski et al. [165]	Cine	2D	2-phase active contour (Balloon + Snake)	Place the initial contour	70 slices	–	Multiphase, SAX + LAX	–						
Lotjonen et al. [166]	Cine	3D	SSM + non-rigid registration	3D surface fitting to create the prior shape model	25(0) subjects	Leave 1 out	Multiphase, SAX + LAX	**2.77** **±** **0.49**	2.01 ± 0.31	**2.77** **±** **0.49**	2.37 ± 0.50	2.56 ± 0.88	2.93 ± 1.30	2.53 ± 0.70
Koikkalainen et al. [167]	–	3D	Artificial training sets enlargement for SSM	3D surface fitting to create the prior shape model	25(0) subjects	Leave 1 out	ED, SAX + LAX	**1.87** **±** **0.63**	1.46 ± 0.30	**1.87** **±** **0.63**	2.26 ± 0.46	2.28 ± 0.63	3.22 ± 1.62	2.06 ± 0.55
Wierzbicki et al. [168]	Cine	3D + T	PCA based template registration + motion extraction	Manual segmentation on training sets	10(0) subjects	Leave 1 out	Multiphase, SAX + LAX	3.4 ± 0.9		**3.5** **±** **1.1**	–	3.2 ± 0.7	3.5 ± 1.1	4.2 ± 1.5
Peters et al. [169]	Cine	3D	Mesh registration + simulated search for boundary detection	3D surface fitting to create the prior shape model	42(42) volumes	Fourfold cross-validation	ED, SAX + LAX	0.83 ± 1.17	0.69 ± 1.13	–	0.74 ± 0.96	0.72 ± 1.14	0.63 ± 0.95	0.76 ± 1.08
Zhuang et al. [170]	Cine	3D	Multi-atlas propagation + refinement + label fusion	Manual segmentation on training sets	37(19) volumes	10 reference shapes	ED, SAX + LAX	2.32 ± 0.82	1.47 ± 0.32	–	2.13 ± 0.70	2.38 ± 1.14	2.22 ± 0.75	2.14 ± 0.63
Zuluaga et al. [171]	Cine	3D	Multi-atlas propagation + refinement + label fusion	Manual segmentation on training sets	22 subjects	Leave 1 out	ED + ES, SAX	DC: LV volume: 0.95; RV volume: 0.92; LA volume: 0.92; RA volume: 0.89; Myocardium: 0.87; Aorta: 0.86						
Zhen et al. [17]	Cine	2D	Multi-output regression with random forest	Manual segmentation on training sets	125 subjects	Leave 1 out	Multiphase, SAX	CC: LV volume: 0.91; LA volume: 0.87; RV volume: 0.88; RA volume: 0.86						

“+T” temporal information is in use, *DC* dice similarity coefficient (ideally 1), *CC* correlation coefficient (ideally 1), *S2S* surface-to-surface distance; 37(19) means 19 out of 37 subjects are abnormal or unhealthy. Figures in bold mean the method takes epicardium of LV and RV as a whole

### The anatomy of the heart

**Fig. 1:**
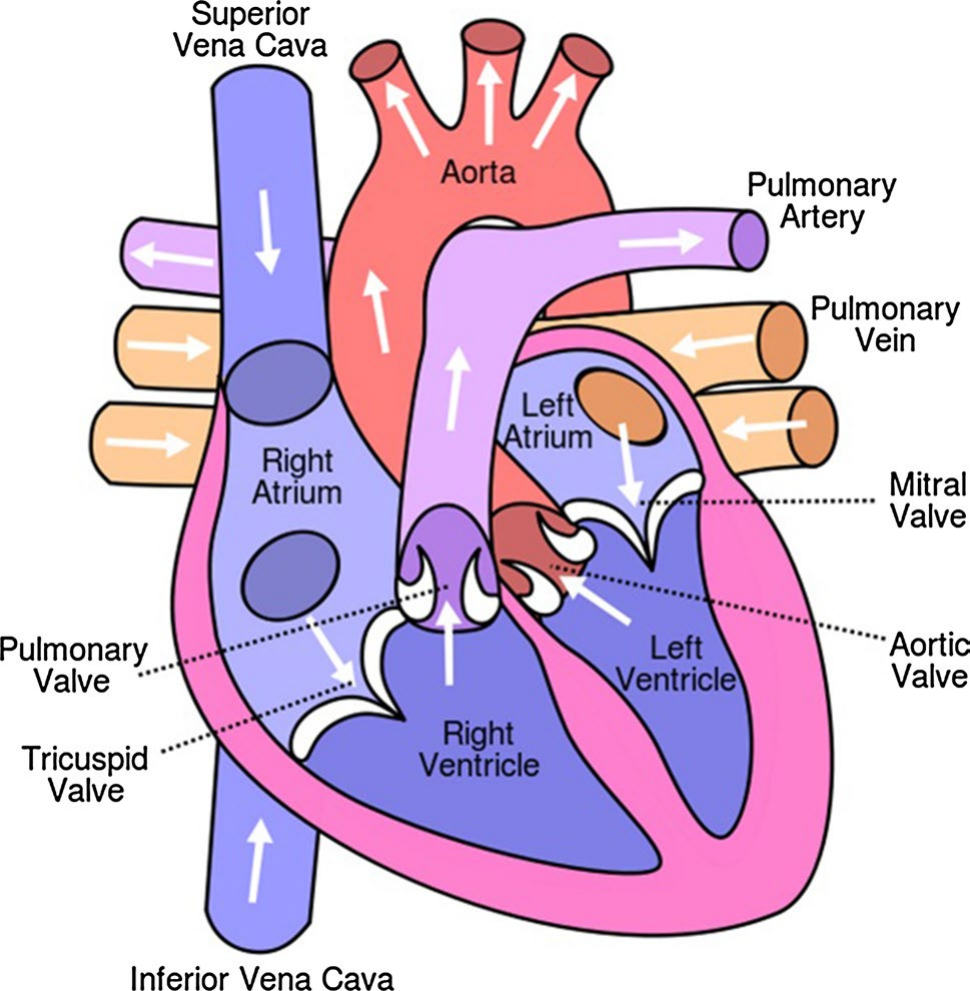
The anatomy of the heart. https://en.wikipedia.org/wiki/Heart

In this section, we briefly describe the anatomy of the heart to help readers establish a better association between the outcomes of various functional analysis methods and the actual structure of the heart (see Fig. 1). Essentially, the heart provides the blood circulation system with indispensable pressure. By contracting and relaxing in turns, it transports blood to different parts of the body through the vessels. The septum separates the heart into two halves that consist of an atrium and a ventricle. The left atrium (LA) and left ventricle (LV) are partitioned by the mitral valve, while the right atrium (RA) and the right ventricle (RV) are partitioned by the tricuspid valve. The semilunar valves are located between the pulmonary artery or the aorta and the ventricle. The RA recycles the low-oxygen blood while the RV delivers it to the lung. After it is oxygenated, the blood flows into the LA, while the LV pumps it to the rest of the body. The myocardium, the muscular tissue of the heart has an inner and outer border: the endocardium and the epicardium, respectively.

### MRI protocols

Since pathological changes are related to abnormal structural and physiological indices, experts are seeking for a more accurate diagnosis or risk stratification of CVDs based on quantitative anatomical or functional information. Various imaging techniques for clinicians have been developed. Unlike radioisotopes, computed tomography, and angiography, CMR is a non-invasive imaging technique that is capable of generating images in decent resolution without ionising radiation. Compared to the traditional echocardiography, CMR does not suffer from speckle artifacts and produces good contrast between the different soft tissues. Images can be obtained in any orientation allowing for images to be acquired in specific anatomical planes. Owing to these properties, scientists have been developing diverse protocols providing varying information. Among them, cine CMR, flow CMR, tagged CMR, late gadolinium enhancement (LGE), and perfusion CMR are the mainstream applications.

**Fig. 2:**
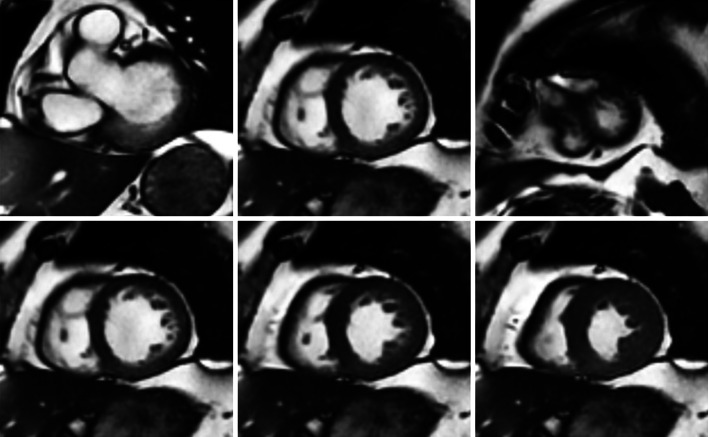
Short-axis cine MR images. *Top row*: slices from base to apex; *bottom row* mid-cavity slice from diastole to systole, displayed using our automatic cardiac segmentation platform GIMIAS. www.gimias.org

**Fig. 3:**
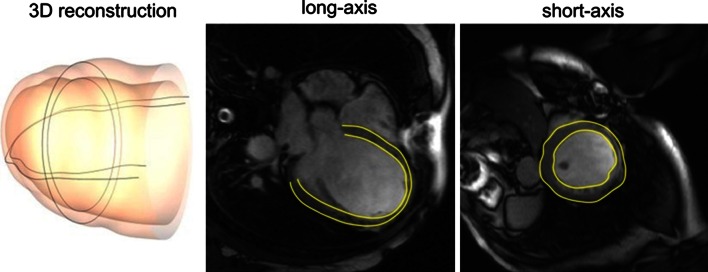
LV segmentation in both long-axis and short-axis views [18]

*Cine CMR* aims at providing fine spatiotemporal resolution with high contrast between the tissues. One sample normally contains 20–30 consecutive frames, corresponding to 20–30 time points in the cardiac cycle. Each frame has multiple slices from base to apex (Fig. 2)—typically between 10 and 15. Generally, the images are captured along two axes: the long axis and the short axis views (Fig. 3). The long axis (LAX) goes across the LV from base to apex. The short-axis (SAX) slices are perpendicular to the LAX. Because the frame sequence loop reflects the dynamic process of a complete cardiac cycle during a breath-hold [19], cine CMR is widely employed in calculating global functional indices such as stroke volume and ejection fraction.

*Flow CMR* is a velocity-encoded protocol, based on the principle that the pulse phase shifts of moving protons are proportional to their velocity along the magnetic field gradient direction [20]. Therefore, the motion of a tissue will generate an MRI signal variation. Flow CMR commences with a reference MRI scan, which uses stationary spins. Afterwards, a number of scans are produced to encode the velocity information by adjusting the direction of the gradient from +180° to −180°. In consequence, moving protons show different intensities from the initial scanning: the brighter areas on phase contrast images are drawn by the protons moving along a certain direction; the darker areas have protons going towards the opposite way; the regions where the stationary protons rest appear to be grey. This property gives flow CMR the advantage in measuring the cardiovascular flow and strain rate.

**Fig. 4:**
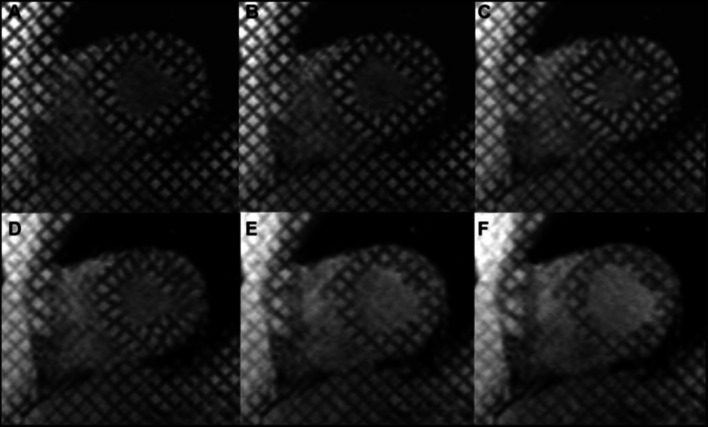
Short-axis tagged MRI mid-cavity slices: **a** tagging produced at end-diastole; **b**–**d** tag lines deform with myocardial contraction in systole; **e**, **f** tag lines deform with myocardial relaxation in diastole; **f** tag lines fade as the end of a complete cycle is approaching [24]

*Tagged CMR* builds a spatial line or grid pattern on the myocardium, which is then followed over the cardiac cycle to estimate cardiac motion. This is based on the received signal from myocardium by modulating saturated magnetisation inside the ventricular wall [21–23]. The dark pattern, which stands at a fixed position on the myocardial tissue, is usually added at end-diastole using radio-frequency excitation and gradient impulses before image acquisition. During the contractile cycle, the dark patterns will move with the tagged tissue, as shown in Fig. 4. By tracking the displacement and distortion of those saturated patterns marked on the tissue, researchers can compute the precise myocardial deformations or reconstruct the wall motion easily. Therefore, tagged CMR is efficient in regional assessments such as for the estimation of myocardial strain and torsion. The limitation of this promising protocol is that the markers always fade inevitably before the whole cycle ends. Also, the existence of the grids brings difficulties to automatic cardiac border identification. The progress and challenges of MRI tagging have been summarised in [23–25].

*Displacement Encoding with Stimulated Echoes* (*DENSE*), which combines the merits of flow CMR and tagged CMR, intends to map the myocardial displacement in high spatial resolution over long periods of cardiac cycles [26] without having serious fading. Different from flow CMR, DENSE uses stimulated echo to modulate the phase, which aims at capturing the emerging displacement between the second and third radio-frequency pulses. This technique can be applied to abnormal contraction diagnosis, myocardium deformation, and motion analysis. However, the imaging is usually time-consuming.

*Strain*-*Encoded* (*SENC*) *CMR* is designed to obtain longitudinal strain straightforwardly, without dealing with displacement or velocity [27]. The dense estimation of longitudinal strain is achieved by processing the tag information extracted from two short-axis images, whose planes are orthogonal to the strain imaging orientation. The tags express the local strain as intensity and their surfaces are set to be parallel to the short-axis images. The short-axis images are generated with two phase-encodings based on slice selection. It has been shown that SENC is a reliable tool to quantify regional myocardial systolic and diastolic function [28].

**Fig. 5:**
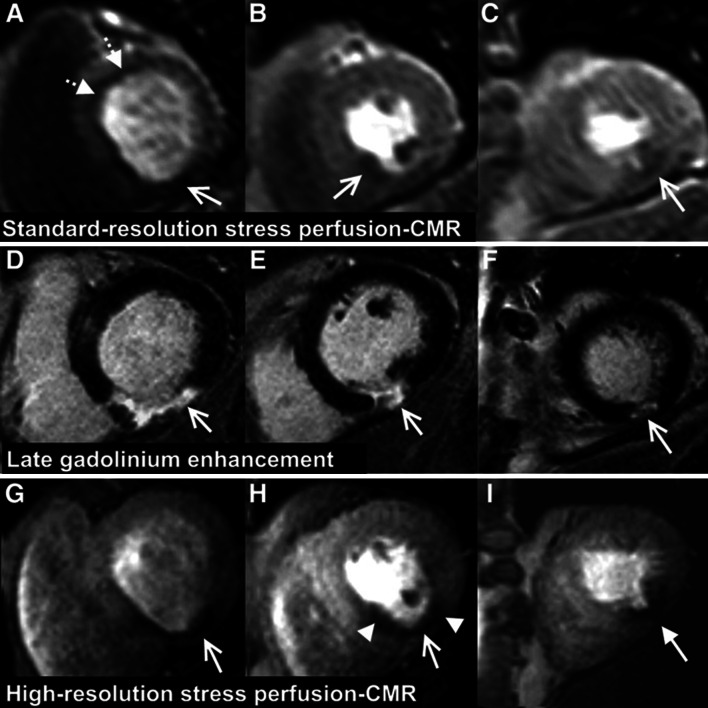
Examples of patients with ischemia acquired in typical late gadolinium enhancement, standard, and high-resolution perfusion MRI. *Arrows* indicate the inferior scar with thinning of the myocardium [30]

*Perfusion CMR* produces contrast-enhanced images by injecting contrast agent (typically gadolinium-based chelates) [29]. The contrast agent travels through the vessels or lymphatic system as the blood flows past and finally reaches the target tissue, which leads to a variation in signal intensity of the agent. A fast scanner with high temporal resolution is responsible for monitoring this signal fluctuation and then sketching sequential images. Perfusion CMR is used for diagnosing ischemic heart disease, for which the myocardium is associated with less blood movement (see Fig. 5). However, perfusion CMR suffers from quantitative analysis degradation introduced by artifacts, ranging from surface coil inhomogeneity, dark rim to motion artifacts. Many researchers have proposed solutions to these inherent weaknesses [10].

*Late Gadolinium Enhancement* (*LGE*) *CMR* is an important technique for the estimation of scar tissue in the myocardium [31]. This technique acquires images (6 mm SAX slice thickness with 4 mm gap for contrast-enhancement match), followed by an injection of 0.10–0.15 mmol/kg intravenous gadolinium [32]. After a delay of 10–20 min, by using the inversion-recovery fast gradient echo (IR-FGE) pulse sequence, LGE images are collected from the same position with a decent spatial resolution. Normally, the contrast agent cannot enter the myocardial cells. In abnormal cases, gadolinium may gather in extracellular space or even break into the cells due to cell membrane rupture. As a consequence, healthy tissues stay dark while infarcted parts appear brighter on the image (see Fig. 5). Therefore, LGE can be very useful in examining injured tissue with infarction or scars.

## Indices of cardiac function

The existing indices of morphology and function can be divided into two categories: global and regional. Global indices include chamber volumes, stroke volume, ejection fraction, cardiac output, and myocardial mass. Regional or local indices cover myocardial wall thickness and thickening. Strain analysis can be either global or local. We have listed in Table 1 many indices, which are frequently used in CMR research, for cardiac structural and functional analysis. We provide the parameter definitions, requirements for calculations, use cases for cardiovascular diseases, and published normal ranges.

### LV quantification

The LV is the most investigated chamber in cardiac segmentation and structural and functional analysis due to its central role in blood circulation. It has relatively thick myocardial tissues that give blood circulation enough pressure. LV parameters can be abnormal in many CVDs, such as in hypertension or after myocardial infarction.

*Left Ventricle End*-*diastolic and End*-*systolic Volumes* (*LVEDV and LVESV*) are measurements of the amount of blood in the chamber, encompassed by the myocardial tissue, when the heart muscle is relaxed (LVEDV) or contracted (LVESV). The contour on the basal slice from the images stack is drawn on the aortic valve cusps level, resulting in an inclusion of the outflow tract as part of the LV volumes. There is no consensus as to whether to include or exclude papillary muscles from the LV blood pool [33, 34, 41–47].

*Left Ventricle Stroke Volume* (*LVSV*) is the amount of blood ejected from the heart during each contraction. LVSV is the difference between the LVEDV and LVESV.

*Left Ventricle Mass* (*LVM*) measures the myocardial tissue. The volume of the myocardium can be obtained by subtracting the endocardial volume from the volume of within the epicardial border. Subsequently, the mass is the product of myocardial volume and the muscle density. LV mass is prognostic in hypertension [48, 49].

*Left Ventricle Ejection Fraction* (*LVEF*) quantifies the quantity of blood pumped out of the heart in each beat as a percentage. It divides the LVSV by the LVEDV. Normal ranges for LVEF are gender- and age-dependent and also dependent on the analysis approach chosen (e.g. include or exclude papillary muscle from LV volumes). Reduced LVEF is a common final pathway in many CVDs (e.g. dilated cardiomyopathy, remodelling after myocardial infarctions). Hyperdynamic LV systolic function as seen by high LVEF can often be seen in LV hypertrophy (e.g. hypertrophic cardiomyopathy) [50].

*Cardiac Output* (*LVCO*) refers to the amount of systemic flow per minute. It can be estimated by multiplying the LVSV with *Heart Rate* (*HR*), which denotes the heartbeat frequency (beats per minute). LVCO is often normalised by the *Body Surface Area* (*BSA*), and then referred to as *Left Ventricle Cardiac Index* (*LVCI*). In patients with congestive heart failure, the LVCO and LVCI are reduced [51].

**Fig. 6:**
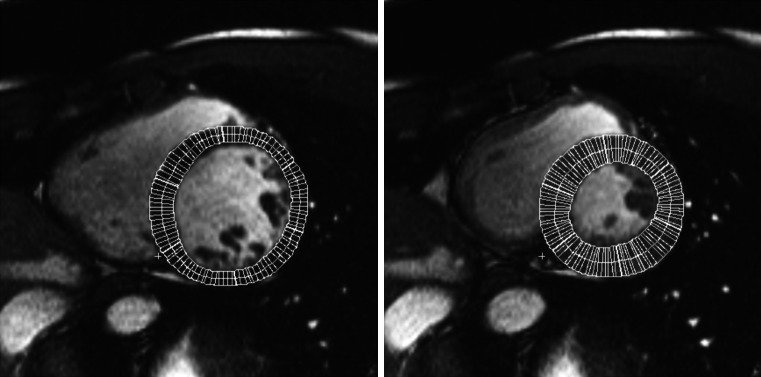
End-diastolic (*left*) and end-systolic (*right*) myocardial wall thickness measurements on LV SAX mid-cavity slices [48]

**Fig. 7:**
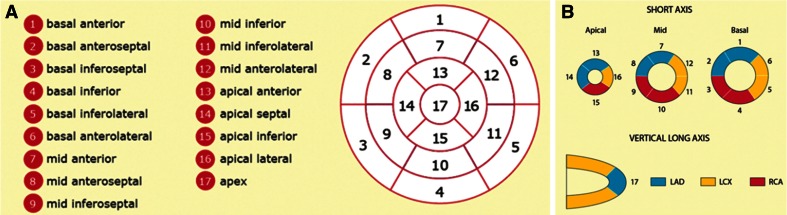
17-segment model: **a** recommended myocardial segments and their nomenclatures on a circumferential polar display; **b** assignment to the territories of the left anterior descending (LAD), right coronary artery (RCA), and the left circumflex coronary artery (LCX). http://www.pharmstresstech.com/stressing/spect.aspx

*Left Ventricle Wall Thickness* is the thickness of the myocardium typically measured on end-diastolic images in SAX view. The papillary muscles and trabecular tissues are usually excluded. First, both epicardial and endocardial boundaries are identified. Afterwards, a centre point or a centreline with reference points is specified to help compute the mean distance between the epicardial and endocardial contours [52], as displayed in Fig. 6. For regional analysis, researchers are encouraged to use the 17-segment model [53] (see Fig. 7). Wall thickness may be globally increased (and hence LVM is typically also increased) in conditions with increased afterload, such as hypertension. Some conditions lead to regional increased wall thickness (with or without increased LVM) typically referred to as showing asymmetric hypertrophy, such as seen in hypertrophic cardiomyopathy. In contrast, myocardial infarctions can lead to regional thinning in the area of infarct as a consequence of cardiac remodelling.

*Left Ventricle Wall Thickening* reflects the change of myocardial wall thickness during systole expressed as a percentage. Wall thickness may be employed to quantify regional dysfunction , such as those seen in myocardial ischemia or after myocardial infarction [54].

*Left Ventricle Strain* (*LVS*) indicates the degree of deformation of the ventricles, while *Left Ventricle Strain Rate* (*LVSR*) is the deformation rate. The required parameters can be received by echocardiography, such as tissue Doppler. Some MRI techniques, for example, SENC, DENSE, and tagging, can be complementary. LVS may play an important role in evaluating myocardial infarction, ischemia, and ventricular dyssynchrony [55].

### RV quantification

The RV consists of the apical body, the inflow tract and the outflow tract. The existing RV functional indices quantify the amount of blood being transported to the lung in different forms. As can be seen from Table 1, the definitions of most RV indices, including *Right Ventricle End*-*diastolic and End*-*systolic Volume* (*RVEDV and RVESV*), *Right Ventricle Stroke Volume* (*RVSV*), *Right Ventricle Ejection Fraction* (*RVEF*), and *Right Ventricle Cardiac Output* (*RVCO*), are fundamentally similar to their LV counterparts. The papillary muscles and trabecular tissues are neglected in endocardial contour depiction. RV volumes may be increased in a number of conditions, including cardiac shunts, certain valve diseases, or pulmonary hypertension [56]. RVEF may also be decreased after myocardial infarctions including parts of the RV. Although efforts have been made to extract the boundaries of endocardium and epicardium simultaneously, the mass is still not regularly evaluated because the myocardial wall of RV is 3–6 times thinner than the wall of LV.

### LA quantification

The LA plays an important role for the modulation of LV blood filling. The LA has a relatively complex geometric structure, surrounded by the aorta, pulmonary veins, and arteries. A dilated LA has prognostic value for cardiovascular death [57], stroke [58], congestive heart failure, and atrial fibrillation [59].

*Left Atrium Volume* (*LAV*) assessed when largest during ventricular systole just before mitral valve opening has been demonstrated as a reliable predictor of cardiovascular outcomes [60], including LV diastolic dysfunction [61], incident atrial fibrillation [62], ischemic stroke [63], hypertrophic cardiomyopathy [64], and lone atrial fibrillation [65]. Similar to the calculation of LV volumes, computational methods can be based on either contiguous summation or geometry assumption. In automatic segmentation, the confluence of pulmonary veins and the LA appendage (area under the mitral valve annulus) are abandoned. Three volumetric parameters, the *maximum LA volume* (*LAV*_*max*_), the *minimum LA volume* (*LAV*_*min*_), and the *pre*-*atrial contraction volume* (*LAV*_*preA*_), are used to investigate *reservoir*, *conduit*, and *booster pump* functions during each cycle.

The reservoir describes the filling of LA in ventricular systole. It is modulated by the LV contraction, RV systolic pressure, LA relaxation, and LA chamber stiffness. When blood flows to the LA from the pulmonary veins, the mitral valve is closed and LAV increases to maximum. *Total Emptying Volume* (*LAEV*) and *Total Emptying Fraction* (*LAEF*) are used to quantify the total amount of blood the LA can pump into the LV.

The conduit function involves LV relaxation and LA afterloads. In early ventricular diastole, LAV grows and the atrial blood is suctioned by the LV. The LA acts like a passive conduit and three indices have been proposed to assess its function: the *Passive Emptying Volume* (*LAPEV*), the *Passive Emptying Fraction* (*LAPEF*), and the *Conduit Volume* (*LACV*), which indicate the amount of blood travelling from the pulmonary veins to LV.

The booster pump, also called contractile function, quantifies the amount of blood being pumped into the LV during LA contraction. It is modulated by the LV compliance, LA afterload, LA preload, and intrinsic LA contractility. In late ventricular diastole, the LA pumps all the remaining blood to the LV actively, and thus the LAV decreases to minimum. Corresponding measurements include *Active Emptying Volume* (*LAAEV*) and *Active Emptying Fraction* (*LAAEF*).

### RA quantification

RA is not routinely assessed in CMR. However, its enlargement may indicate heart failure, as well as valvular and congenital diseases [40]. The filling of the RA is related to the functions of the RV. *RA volume* (*RAV*) indexed to BSA can predict pulmonary hypertension [66, 67] and chronic systolic heart failure [68]. Because direct measurement of RAV can be time-consuming, scientists primarily do the estimation based on single or bi-plane area-length methods through inspecting RA areas on two-chamber and four-chamber LAX viewing (see Table 1).

## Cardiac segmentation

In order to calculate the CMR structural and functional indices listed in this section, the boundaries of the heart chambers are necessary. However, delineating the heart manually on multiple slices and frames requires lots of time. Furthermore, this is subject to well-established intra- and inter-subject variability. This has motivated engineers to develop automated cardiac segmentation techniques that can rapidly, objectively, and accurately extract the chamber boundaries from CMR in clinical practice. Although MRI provides decent soft tissue contrast among different protocols, accurate cardiac segmentation remains a great challenge for the researchers due to inevitable imaging inhomogeneity and high anatomical variability, as well to the inherent geometric and dynamic complexity of the heart. In this section, we will describe the existing segmentation methods published in popular journals and conferences from the year 2000, by focusing on their principles, functions, advantages, and limitations.

### Segmentation methodologies

Generally, semi-automatic or fully automatic segmentation techniques fall into two categories: (1) image-driven approaches without or with weak prior models and (2) model-driven approaches based on strong prior knowledge. Training data are examples with ground-truth. Image-driven methods identify the pixels or voxels belonging to the blood pool, myocardium, or appendage by visiting their intensity differences. Typical image-driven techniques consist of thresholding, region-growing, clustering, pixel or voxel classification, and active contour or surface. Strong prior knowledge, including cardiac atlases and statistical shape models (SSMs), make use of statistical information extracted from manually annotated training data that describe for example averages and modes of variations of the cardiac chambers. For the rest of this section, we briefly discuss each of these techniques.

*Thresholding* can be used to localise the region of interest (ROI), such as the blood pool or myocardium, based on analysing the intensity histogram. The latter is usually constructed as a discrete distribution of pixel intensities (counts vs. values). Then a threshold value, which corresponds to a specific intensity, is to divide the histogram into sub-intervals containing distinctive modes. The pixels having intensities in a same interval may belong to a certain type of tissue. This method is only effective when significant intensity diversity exists between the target and background areas. However, in some cases, the intensity of different tissue types overlap. Therefore, thresholding is often used as a pre-processing step and further combined with other segmentation techniques.

*Region*-*growing* starts with choosing one or multiple seed points in MR images in a selected region such as the myocardium. Afterwards, the initial region begins to grow by searching similar pixels nearby or inside a neighbourhood. If a pixel (*x*, *y*) meets the designed criterion, it will be allocated to region *R*_*i*_ in the *i*th step: *R*_*i*+1 _= *R*_*i*_ ∪ (*x*, *y*). When none of the surrounding pixels qualify, the region stops growing as it may have reached the boundary of the tissue. *Merge* behaves alike, but instead of judging single pixels, it combines similar small regions. While *split* performs in the opposite way, it shatters the region or suspends the membership if a sub area differs significantly from the rest of the area. Because of the continuity of growth, region-growing, or split and merge techniques, can often lead into over segmented target tissues, leaking into fragments of irrelevant parts. For instance, the aorta and cavity may have close intensities on basal SAX slices and cannot be distinguished using only thresholding. Watershed [69] combines thresholding and region merging by calculating the image gradient map and setting a threshold on the magnitude of the gradients. If a pixel and its adjacent neighbours all have similar magnitudes below the specified threshold, they are merged. Watershed is known to result in over-segmentation and poor performance in noisy regions due to the reliance on image gradient.

*Pixel or voxel classification* groups pixels in 2D or voxels in 3D in feature space. Patch-based features contain pixel intensity or textural appearance information. Unsupervised clustering, which is non-parametric, does not require manually labelled training data. Typical methods include *K*-means clustering and expectation–maximization (EM) [70]. *K*-means clustering randomly chooses *K* features as the initial centroids and classifies all other features according to their distances to the centroids, then calculates new centroids of those categories. These steps are repeated until centroids are converged and no longer change. EM finds the maximum likelihood (ML) or maximum a posteriori (MAP) estimates of parameters of a statistical model. For cardiac segmentation, a common model is the Gaussian Mixture Model (GMM), in which each tissue histogram follows a Gaussian distribution. Every pixel is classified to the region that maximises its corresponding class conditional probability. Supervised classifiers, such as *K*-nearest neighbour (KNN), random forest, and neural network, need manually labelled training data. In these methods, the training data and their associated labels are regarded as examples from which the parameters of the classifiers are learned by minimising a risk function that pertains to misclassification of the training labels. Each test pixel or voxel can be accordingly classified afterwards using the learned classifiers. However, annotating training data involves user interaction and the performance of these classifiers often depends on the quality of training samples. If the training and testing datasets statistically deviate by a large extent, the classification performance declines significantly. Moreover, classification-based segmentation methods often ignore the spatial dependencies of the local features. The advantage of supervised techniques is that they are trainable to segment more accurately, provided that the expert knowledge is properly employed in the classifier.

*Active contour*-based methods or *snakes* [71] search for chamber walls, instead of directly classifying the regions. A curve parametrised *C*(*s*) = (*x*(*s*), *y*(*s*)) where *s* denotes a free parameter, is morphed locally towards target boundaries by minimising a predefined energy. In order to achieve a better result, many researchers have designed different energy functions. Generally, the energy *E* can be written as $E = \smallint E_{\text{in}} (C(s)) + E_{\text{ex}} (C(s)) + E_{\text{c}} (C(s)){\text{d}}s$, where *E*_in_ indicates the internal force that aims at retaining the topology and smoothness of the curve, *E*_ex_ is the external force pushing the curve to target boundary and *E*_c_ stands for additional constraints. The last aims at improving convergence or penalising unwanted shape irregularities. Segmentation based on active contour may need user interaction, for example, roughly drawing or placing a contour for initialisation. An improvement over the traditional form of active contour is achieved using level-set formulation, in which the curve implicitly defined as the zero level-set of a higher dimension function [72–74]. The level-set can handle larger shape updates, when the morphology of the curve has to be evolved significantly. To segment the hearts in a multi-phase fashion, the converged segmentation can be propagated into images in the subsequent time points for a better initialisation, removing user interaction.

*Direct estimation* has been recently proposed as a means to estimate functional indices such as chamber volumes without segmenting MRI slices [14–17]. This approach has proved to be an efficient tool in myocardial abnormalities detection [14]. It uses regression-like models trained with discriminative image representations to estimate the ventricle volumes from image information. Different from the pixel or voxel classification problem, the whole or parts of the image act as global input features to establish similarities to reference samples with known functional indices.

*Atlas*-*based* segmentation methods rely on the spatial probability patterns of various tissue types of a typical heart. To segment a test case, the image is registered to the atlas, which serves as the prior information for the pixel labels given their locations. It has been demonstrated that multi-atlas-based segmentation methods outperform single-atlas approaches remarkably in terms of accuracy in other applications [75–79].

*Statistical shape modelling* introduced by Cootes et al. [80], is a powerful tool for cardiac quantitative assessment. Given a population of corresponding points or vertices from myocardial surface meshes, a mean shape is extracted and a set of variation modes can be built using principle component analysis (PCA). Then any novel shape from an individual can be represented as the mean shape varied by a linear weighted combination form of the PCA modes. This representation is called the point distribution model (PDM). For segmentation based on ASM [80] or AAM [81], the linear model is matched to the test image by matching landmark through global transform and finding an optimal loading vector of PCA modes. This is usually achieved iteratively, updating one set of parameters at a time.

Despite popularity of ASM/AAM-based segmentation approaches, their training demands identifying a dense set of corresponding landmarks over the training population. Furthermore, the fitting procedure can be computationally slow and prone to local minimums. Models constructed from healthy populations many not fit pathological hearts well, as the model becomes too restrictive. Such large inter-class variation provokes a need for training more generalizable models, as well as more sophisticated fitting processes. Nevertheless, model-based methods are still considered to be promising routes to accurate MRI segmentation as they are capable of preserving anatomical spatial knowledge while segmenting the heart. In the following sections, we detail the specific roles and efficacies of these techniques in quantitative analysis of LV, RV, LA, bi-ventricle, and whole heart segmentation.

### LV

Among various compartments of the heart, LV has been studied the most extensively, as it pumps the blood into other parts of the body. A relatively thick myocardial wall leads to the popularity in the research of regional assessment, such as apical, middle, or basal wall thickness, local deformation, and myocardial strain. Only those approaches that process multiple phases (including end-diastole and end-systole) can measure *LVEF*, *LVSV*, and *LVCO*, since *LVEDV* and *LVESV* are known.

Methods able to extract both the endocardium and epicardium can be used to calculate the *LVM* and *wall thickness*. For *wall thickening*, the centreline method performs better, as its radial opponent often overestimates the distances between the contours of epicardium and endocardium. This is caused by the initial hypothesis of the radial method, which assumes the shape of myocardium as a circle [82].

*LVS* can be analysed by tracking myocardial motion, since regional muscular displacement and temporal information are both required. The global strain analysis in 3D begins with creating Cartesian coordinates. The extent of deformation, described as the change of length from an initial or reference status, can be calculated using Lagrangian or Eulerian formulae [83]. Because the heart deforms along different directions in Cartesian coordinates in 3D simultaneously, a matrix called a tensor is created to describe the process. For regional analysis, the local coordinates are with three mutually perpendicular axes: the radial (perpendicular to the epicardium and towards the outside), the longitudinal (tangent to the epicardium and towards the base), and the circumferential (according to the right-hand rule, from radius to longitude) axes. Therefore, the spatial orientation of three axes varies with the voxel position in the myocardium.

Theoretically, *LV volumes* can be estimated with any 2D segmentation outcome on SAX or LAX slices, not necessarily 3D, by making use of provided volumetric calculation methods in Table 1. However, when only SAX slices are in use, the segmentation must be completed on a stack of multiple slices from the base to the apex. We list all the LV segmentation techniques described in this section in Table 2.

#### Thresholding and region-growing

**Fig. 8:**
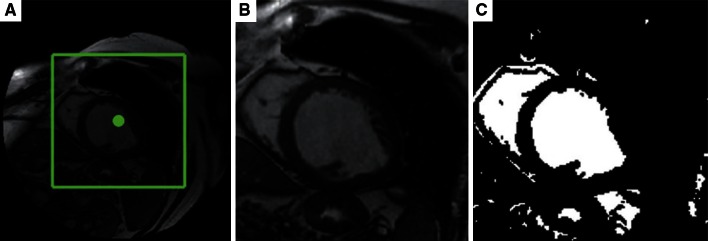
LV endocardium delineation using thresholding: **a** detected region of interest (ROI); **b** ROI image; **c** converted binary image using optimal thresholding [87]

Thresholding is often integrated with region-growing. Lee et al. [84] and Codella et al. [85] use region-growing to find the full-blood LV region. They automatically identify a seed point by taking the pixel with the lowest energy in a window during slice propagation. Then in order to prevent the segmented LV region from diffusing to epicardial fat, fluids, and RV, they use an iterative thresholding mechanism that discovers a lower bound of myocardial intensity. Huang et al. [86] employ thresholding to distinguish the blood pool from the myocardium, followed by radial region-growing and extraction of convex hulling to identify the endocardial and epicardial boundaries. Lu et al. [87] apply thresholding to convert a ROI to a binary image for LV localisation and endocardial contour detection (Fig. 8), followed by region-growing to segment the LV epicardium. Ammar et al. [88] take the binary image produced by thresholding as the initial mask for a level-set segmentation method to extract the endocardium. Queiros et al. [89] perform class decomposition following thresholding step to search for the LV centroid. The method sets two thresholds for myocardium and cavity histogram in an EM algorithm to extract the endocardial contour. Kurkure et al. [90] localise LV in the thresholded image by finding a binary component that is closest to the intersection cross-hair generated by LAX vertical and four-chamber view projection in ED phase on a SAX slice. They have also proposed a novel fuzzy connectedness region-growing method taking the spatial adjacency, intensity homogeneity, and multiclass features into consideration. The myocardial boundaries are extracted by dynamic programming, which is an optimal path finding solution of overcoming obstacles such as papillary muscles or trabeculae carneae extrusion, and low liver-to-myocardium tissue contrast. Cousty et al. [91] extract epicardium by developing a spatial–temporal gradient computation for watershed cuts. It is noteworthy that approaches in [84–87, 89] start their segmentation from mid-ventricular SAX slices, which might involve user interaction, and then propagate their initial results to other slices as prior knowledge. Also, the test image is usually mapped to the polar coordinate since LV roughly has a circular shape [84–87, 89, 90]. Furthermore, by making use of thresholding on the LV blood pool, the papillary muscles and the trabeculations can be easily outlined [84–87] due to their intensity diversity with surroundings. Thus, cardiac functional analysis such as *LVEDV*, *LVESV*, or *LVM* estimation be can varied by including or excluding papillary muscles and trabeculations or not, depending on the index definition and requirement of clinicians.

#### Pixel or voxel classification

**Fig. 9:**
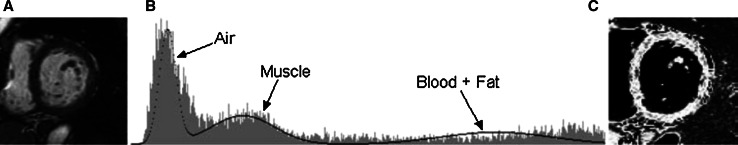
Pixel classification by fitting a Gaussian Mixture Model to the histogram of the input image: **a** the input short-axis image; **b** 3 Gaussian distributed components representing the air, myocardial muscle, and blood/fat compartment; **c** the output image with classified pixels in different labels [92]

Classification-based methods in cardiac segmentation have also been thoroughly studied. Jolly [92] and Hu et al. [93] propose to classify regions by a 3-GMM with EM, based on the intensity histograms. Jolly [92] separates the muscle, air, blood, and fat, as presented in Fig. 9. Hu et al. [93] separate the muscle, the blood and the background. Pednekar et al. [94] fit a 5-GMM to the intensity histogram of the blood pool, the lung filled with air, myocardium, the region between the blood and myocardium, and the region between the air and myocardium. The EM algorithm is initialised through *K*-means clustering. Queiros et al. [89] use a 2-GMM. Some classifiers label the features from different tissues without making assumptions on intensity histogram distribution. To label the regions of the lung, the myocardium, and the blood pool, Stalidis et al. [95] make use of a neural network classifier, which is trained via a small number of representative tissue points. The input features of their classifier are the pixel position, pixel intensity, and slice location. Folkesson et al. [96] have presented that a trained KNN classifier is competent for the classification of the LV cavity, myocardium, and background, based on a feature selection scheme. The latter finds the most discriminative features for the pixel classifier or model fitting, aiming at increasing computational efficiency without degrading its accuracy. Bai et al. [97] have shown that the support vector machine (SVM) outperforms KNN in label fusion in a multi-atlas-based cardiac segmentation framework.

#### Active contours

**Fig. 10:**
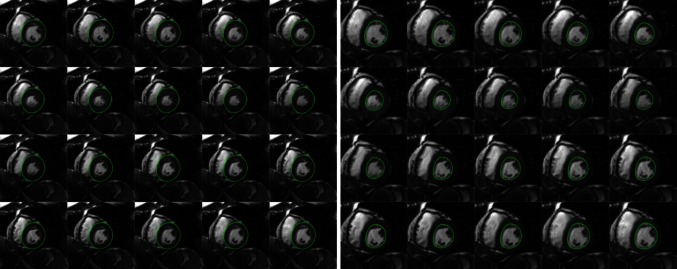
LV epicardium (*left*) and endocardium (*right*) tracking: contours propagate through short-axis slices on all phases in a complete cardiac cycle [106]

**Fig. 11:**
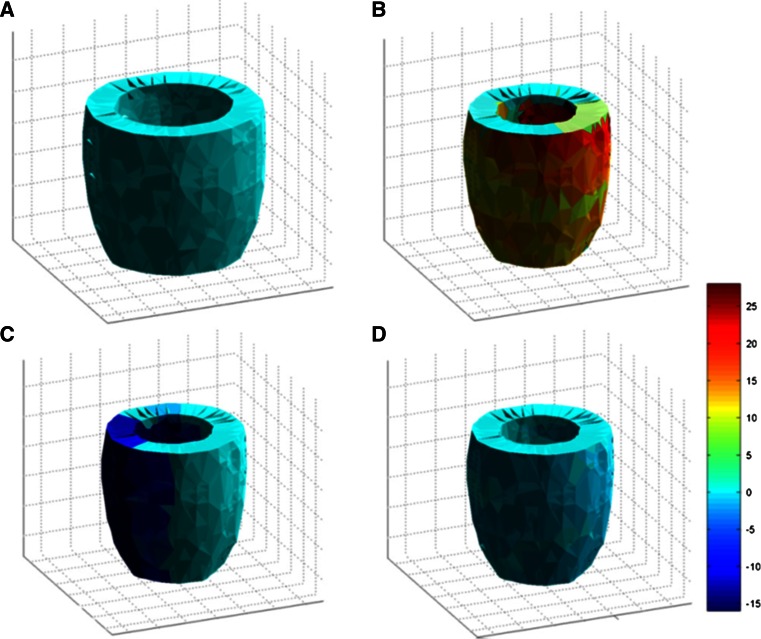
Examples of detected LV myocardial strains visualised in 3D: **a** ED strain; **b** ES radial strain; **c** ES circumferential strain; **d** ES longitudinal strain [115]

Active contour, or deformable model, is one of the most widely applied techniques in heart segmentation. The breakthrough usually comes with the design of the energy function, such as using anatomical assumptions as the constraints on the level-set methods [98, 99]. Paragios [98] propagates coupled endocardial and epicardial contours on SAX slices, where the edge, region, and anatomical constraints are pre-defined. The edge constraint is used to push the curves to the myocardial walls. The region intensity criterion makes the model less sensitive to initial conditions. The GVF snake, a parametric active contour that overcomes the difficulty in evolving the curve to the boundary concavities, is introduced into LV segmentation in these works [100–102]. Wu et al. [103] claim that the gradient vector convolution (GVC) snake also conquers local minima such as artifacts and papillary muscles. Kaus et al. [104] integrate strong prior knowledge, in the form of PDM, into deformable contours by extending their internal energy, leading to an increase in the robustness of the model. They have considered the inter-spatial relationship of the inner and outer boundaries as well, which compensates for the error produced by incorrect feature detection. Lynch et al. [105] employ the probability density function as the prior information, which is created by a set of manually segmented boundaries on binary images. Furthermore, the evolutions of endocardial and epicardial curves are coupled by an extra level-set constraint. The deformable models proposed in [106, 107] and the level-set approaches presented in [108, 109] incorporate the spatial–temporal LV activation as prior knowledge and track the epicardium/endocardium boundaries on SAX slices in a complete cycle. A typical tracking result is shown in Fig. 10. The constraints in [106, 107] are parameterised by Fourier descriptors. Moreover, an approach for the recognition of intra-ventricular dyssynchrony (IVD) is proposed in [106], where the non-uniform contraction of the ventricular walls brought by the activation delays can be discovered. Jolly [92] makes use of a deformable model to improve further the outcome of EM-based region segmentation. Chen et al. [110] propose to apply deformable models to *LVS* analysis on SAX slices in DENSE MRI. Their model is driven by minimising an energy function that consists of model intensity, edge attraction, shape prior, contours interaction, and smoothness. The shape prior can eliminate the concavities with negative curvatures in order to remove the papillary muscles from the ventricular walls. Huang et al. [111] have invented a novel deformable model called Metamorphs, whose energy functions are predefined on the distance maps of the object shape and its border. Metamorphs is not particularly designed for MRI cardiac segmentation while it outperforms the GVF snake as a result of better robustness to inferior initial conditions. Based on the motion trajectories in DENSE, representing the movement of myocardial wall between two consecutive phases, the initial manually drawn endocardial and epicardial contours can be propagated slice by slice to other frames [112, 113]. The method proposed in [112] is applicable to both SAX and LAX images. Besides DENSE, Chen et al. [114] have also used tagged MRI to derive *LVS*. In their work, Gabor filters search the tag intersections. Through matching these intersections, the method is able to track the myocardial motion. The deformable model refines the tracking and displays a dense displacement map. Kermani et al. [115] draw a dense displacement map by fitting a 3D active surface model to an initial sparse displacement map, which is built by establishing point correspondence in cine images. Motion tracking makes *LVS* easier to be analysed, because the myocardial displacement and temporal scale are known at the meantime. The authors produce visualisations of LV strains in 3D (see Fig. 11). Khalifa et al. [116] measure the *wall thickness* and *thickening* with a stochastic speed function based level-set technique extracting inner and outer myocardial walls first. Subsequently, the points on the inner contour and the outer contour are paired. The Euclidean distance between each pair is the *wall thickness*. Wei et al. [117] use the myocardium contours from cine MRI as prior knowledge to guide the meshing of endocardium and epicardium, which are generated by contour registration to move towards the inner and outer edges in SAX and LAX slices in LGE. Grande et al. [118] model the image likelihood by sampling the intensity and gradient of pixels inside the myocardium or at the boundary of myocardium in different regions. After that, they create a Markov Random Field (MRF) to incorporate the prior and the likelihood models. The prior keeps the curve smooth and excludes the papillary muscles. The deformable model estimates the walls based on the MRF along the SAX radial direction.

#### Strong prior based techniques

**Fig. 12:**
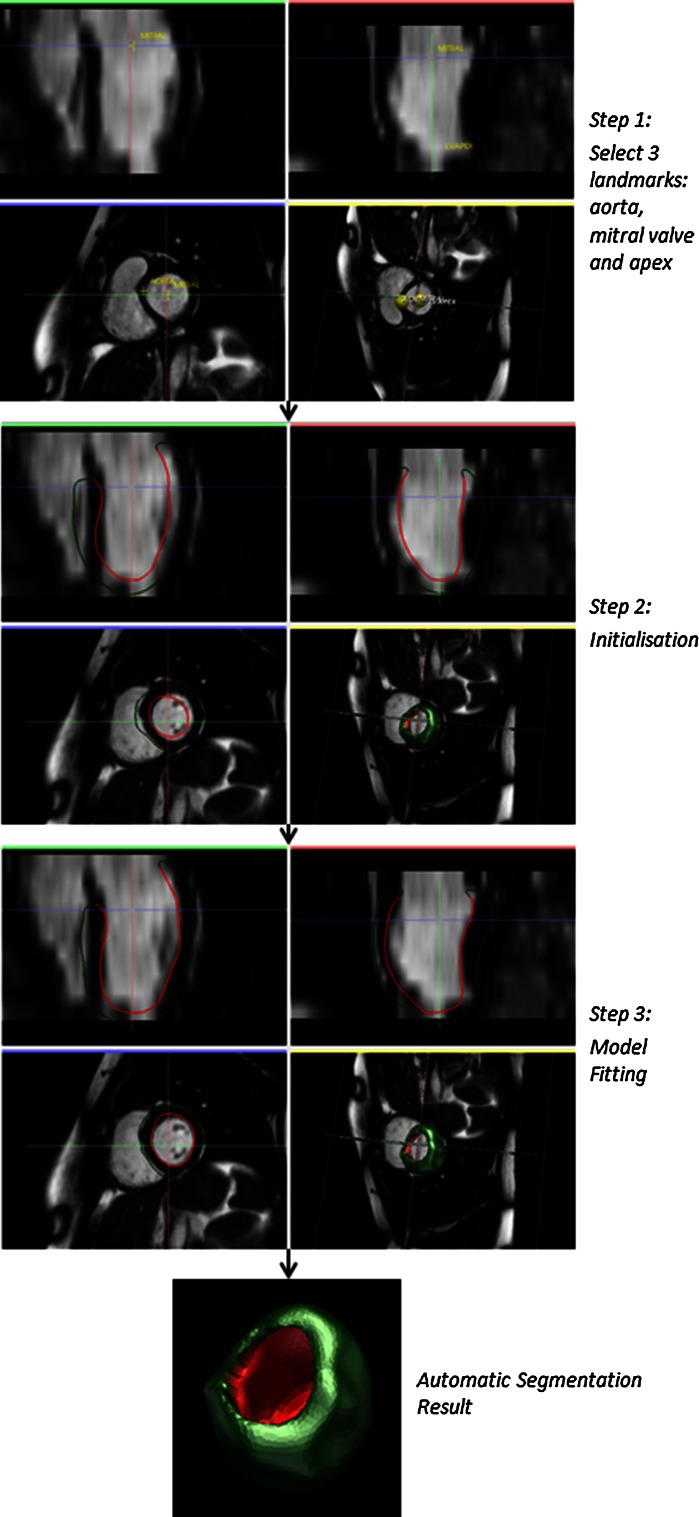
A 3D-ASM (SPASM) LV segmentation technique [120] using GIMIAS platform: *Step 1* user specifies three landmarks (the aorta, the mitral valve, and the apex) by three clicks on the cine MR volumes; *Step 2* the platform automatically generates a model (a triangular surface mesh), which is pre-constructed in training stage, based on the three given landmarks; *Step 3* the model fits to the target (feature point detected via fuzzy inference) through propagating the updates from the vertices close to the intersections between the surface and the image planes to distant regions on the earth

Different from image-driven techniques, model-based approaches exploit strong prior knowledge such as by encoding the specific shape variability of the LV, instead of making simple assumptions on the boundaries. By taking advantage of the statistical shape information, segmentation becomes more robust to image noise by restricting the outcome to valid instances statistically. Mitchell et al. [119] introduced early application of 3D-AAM to LV segmentation in 2002. The method showed worthy results in quantifying *LVV*_epi_, *LVV*_endo_ , and *LVM* on SAX volumes. Assen et al. [120] proposed a 3D-ASM segmentation method (SPASM) that can operate on sparse MR images scanned in arbitrary orientations. In most cases, the automated LV segmentation approaches require a stack of parallel SAX images. While SPASM can perform on a datasets of two orthogonal radial LAX slices, four radial LAX slices with a 45-degree angle between two neighbours, 11 equally spaced SAX slices, four SAX slices (one apical, one mid-cavity, two basal), or a combination of two LAX and two SAX slices. The processing pipeline of this method is implemented and shown in Fig. 12. Lekadir et al. [121] improve the 3D-ASM by incorporating an additional shape prior, which is invariant to transforms including translation, rotation, and scaling. This prior is used to detect and correct outliers, thus leading to more robust results. Andreopoulos and Tsotsos [122] use a hierarchical 2D-ASM that incorporates temporal constraints to enhance the fitting outcome of 3D-AAM. Assen et al. [123] replace the absolute intensity 3D-ASM with relative grey scales when ROI is being identified (fuzzy inference). Suinesiaputra et al. [124] propose to employ independent component analysis (ICA) [125] instead of PCA in SSM to extract myocardial contraction from SAX slices. Furthermore, due to the better performance on local description, ICA is used to design a classifier able to detect regional wall motion abnormalities. Lekadir et al. [126] have also assessed myocardial motion through decomposing the global ventricular shape. They calculate the relationships between a series of spatiotemporal inter-landmarks. By tracking the epicardium and endocardium a dysfunction map is drawn to show abnormal contractions. O’Brien et al. [127] model shape, spatial, and temporal variation separately. They use a global contour optimisation instead of conventional ASM fitting. Roohi and Zoroofi [128] propose a kernel PCA (KPCA), in which the modes applied to represent a global ventricle shape are combined non-linearly. The distribution of landmarks is divided into intra- and inter-subspaces. A more recent work proposes to collect all the shapes learned from training data to build a dictionary [129]. The features of segmented frames from the test image are also added to the dictionary to create a patient-specific model dynamically. Each feature is classified into object (myocardial boundaries) or background (blood pool or muscles). A sparse shape model is then used to find the points on the ventricle walls based on their distances to the classified features. Unreliable points are abandoned and the complete LV shape is reconstructed according to the dictionary. Temporal constraints are not considered in this approach as current segmentation relies on the outcomes of the previous frame. Zhu et al. [130] developed a subject specific dynamical model that simultaneously handles inter- and intra-subject variabilities in a recursive Bayesian framework and a combined multi-linear PCA-ICA model. Starting from a manually segmented first frame, subsequent frames are segmented according to the current intensity pattern and a shape prior, predicted from the past frames.

Xenia et al. [131] proposed a framework for LV segmentation that is based on heuristic rules such as the brightness of the blood pool, sphericity of LV, and inter-slice smoothness of segmentation. A graph-cut algorithm was presented to infer the labels of the myocardium for robust optimization; however, the three-dimensional morphology of the heart was not fully exploited. A two-dimensional segmentation framework was proposed in [132] that effectively maps the edge patterns of each slice (centred at LV centroid) from polar into a Cartesian grid. Then a dynamic programming method is used to walk through the grid having the strongest edge values. Ayed et al. [133] propose another 2D segmentation framework that firstly learns intensity and the shape distributions for the blood pool from a manually segmented frame. Then, using a max-flow algorithm, it minimises a lower bound on the Battacharyya distance between the trained and subsequent distributions obtained from other frames. Similarly, Nambakhsh et al. [134] consider learning both intensity and shape constraints from a segmented first frame, and then minimise the distance between the test and trained distributions. However, rather than a graph-cut based method, a series of convex cost functions are solved for exact minimization. Yet, another training based LV segmentation method is proposed by Eslami et al. [135]; the prior information is implemented through kernel based approach, where test image is compared to the training data for the closest neighbour using a random walk paradigm. The method is shown to segment pathological hearts, whose data is usually overlooked using conventional PCA based statistical shape models.

#### Direct estimation

Afshin et al. [14] propose a direct estimation on heart abnormality detection. In their work, each subject comprises three SAX slices, whereas the apical slice is divided into four segments and the mid-ventricular and basal slices are divided into six segments. This user-provided segmentation is only needed in a single frame, which acts as a reference. All other subsequent frames in a complete cycle are automatically divided into 16 segments each, according to their distribution similarity. The local statistical descriptors, whose dimensions are reduced through linear discriminant analysis (LDA), are then constructed based on these segments. Because each segment can reflect the portion of blood filling, their statistics correlate well with the regional LV function. As a result, with a linear SVM trained by the ground-truth given by the radiologists and features from these local LDAs, regional abnormalities can be detected. They have classified 58 subjects (21 normal and 37 abnormal) with an accuracy of 86.09 %. Direct estimation for cardiac functional analysis can be a promising research direction and has achieved competitive performances in comparison with the state-of-the-art segmentation based assessment, with a significantly lower computational complexity.

### RV

In the literature, the RV has received far less study compared to the LV, due to its more complex shape (in particular the variation of the complex crescent shape from the base to the apex), its thinner walls, and the similar intensity appearance with the trabeculations [11]. Because of these complexities, quantifying the regional myocardium wall thickness is not generally recommended for the MRI-based cardiac functional analysis of the RV. We discuss the available methodologies in the following and list them in Table 3.

#### Image-driven techniques

Maier et al. [136] segment the RV in MRI combining watershed filtering with graph-cut based region merging. They provide two initialisation options: the user either outlines the RV wall in 4–5 slices of the ED phase, or marks two points on the basal slice to register an atlas. Wang et al. [137] use a morphology-based algorithm, which considers the layout, shape, size, and relative locations to locate roughly the LV and RV first. The temporal discrepancy between two consecutive frames is then used to discover RV as the most active part. Ringenberg et al. [138] segment the endocardium by intersecting two ROI constrained binary images as follows. Firstly, an ROI window is selected and converted into binary with an optimal thresholding; next, the same ROI is convolved with a difference of Gaussian filter and thresholded at zero. The RV mask is roughly estimated as the intersection of these images. The window constraints label information from the previous slices and work as prior knowledge. The segmentation begins from the most basal slice in ED and ends at the apex. For ES, the prior is the union of labels from the previous slices at ES and the label of the current slice at ED. Punithakumar et al. [139] base their segmentation on registration and propagation. A 2D mesh delineating the endocardium or epicardium moves across all phases by establishing point-to-point correspondences. The manual segmentation of a single frame is required for initialisation. Mahapatra [140] uses a trained random forest classifier to give voxels two probability values, corresponding to the object and background. Based on this probabilistic map, a final segmentation is achieved by graph-cut. The image features they extracted for the discriminative description consist of intensity statistics, spatial context, textural, and curvature entropy. Nambakhsh et al. [141] propose a method based on the global shape and intensity similarity estimation. Based on the global distribution matching, the shape prior is intrinsically invariant with respect to translation and rotations. Centroid of LV and a small area of RV cavity have to be specified by the user. Compared to the learning-based approach in [140], this algorithm has the advantage of requiring only a single subject for training.

#### Model-driven techniques

Because of the geometrical complexity of the RV, robustness becomes a major concern in its automated segmentation. Amongst existing techniques, addressing this challenge, multi-atlas-based methods with label fusion [142–144] have received significant attention. In these frameworks, finding reliable correspondences between the patient and atlas spaces becomes critically important. For instance, Ou et al. [142] present a deformable registration algorithm that uses saliency of the matching for improved robustness versus variation of shape, intensity, and field of view. A “zoom-in” mechanism that uses the first round of RV segmentation to iteratively refine the registration and segmentation outcomes is employed. Alternatively, Grosgeorge et al. [145] employ a PCA-based SSM as the prior model, to guide the segmentation through a graph-cut method. The model is registered to the test case through a rigid transform, with two anatomical landmarks manually placed by the user on the ventricular septum. Oghli et al. [146] apply PCA on signed distance functions extracted from parametrised training contours as the shape prior for a deformable model. In addition, they use region and boundary based energies for improved fitting.

### Bi-ventricle

Bi-ventricular segmentation uses slices covering both ventricles, from apex to the ventricular base, which is the valve plane, for full delineation of LV/RV myocardium. This research area has also been actively explored in the past 15 years (see Table 4). An overview of these methods is covered in this section.

#### Image-driven techniques

Sermesant et al. [147] have proposed a deformable biomechanical model based on tetrahedral geometric representation. The user specifies a proper mesh size to keep the data amount reasonable and retain a good mesh quality. The mesh is mapped to the test image using a non-rigid registration under influence of the internal and external forces, modelling elastic and imaging constraints, respectively. This method can be used for motion tracking, thus measuring local deformation and *LVS* is made feasible. Rougon et al. [148] employ a non-rigid registration method to assess myocardial contraction in both SAX and LAX slices. They use tagged MRI to infer the intra-myocardial motion and the cine MRI to extract the myocardial anatomy dynamically. Hautvast et al. [149] suggest a contour propagation scheme from ED to ES images. This method can be applied to SAX, two-chamber or four-chamber LAX slices, but requires manual segmentation on ED for initialisation. Cocosco et al. [150] convert a test image into a binary representation by optimally thresholding the intensity histogram of the ROI. The fat around ventricles is then removed by a thinning operation. All connected components are labelled and region-growing is performed on the SAX slices. Afterwards, they calculate the volume of each component at all frames and the maximum and minimum values are taken out. Two components having the most significant differences between their maximum and minimum volumes are selected as the LV and RV. The final delineation is obtained by merging the voxels classified in the first step along the LAX direction. Grosgeorge et al. [151] use the seminal model of active contours without edges [152] for bi-ventricular segmentation of a large dataset containing 1920 MR images, and obtained satisfactory results comparable to the state-of-the-art. Mahapatra et al. [153] segment bi-ventricles using a graph-cut framework, guided by a shape prior based on the distribution of orientation angles from each pixel to the edge points, as extracted from a single manually annotated image. Wang et al. [154] adaptively use reinforcement learning to assimilate the knowledge provided by the user, such as edge point position correction, in LV/RV segmentation.

#### Model-driven techniques

**Fig. 13:**
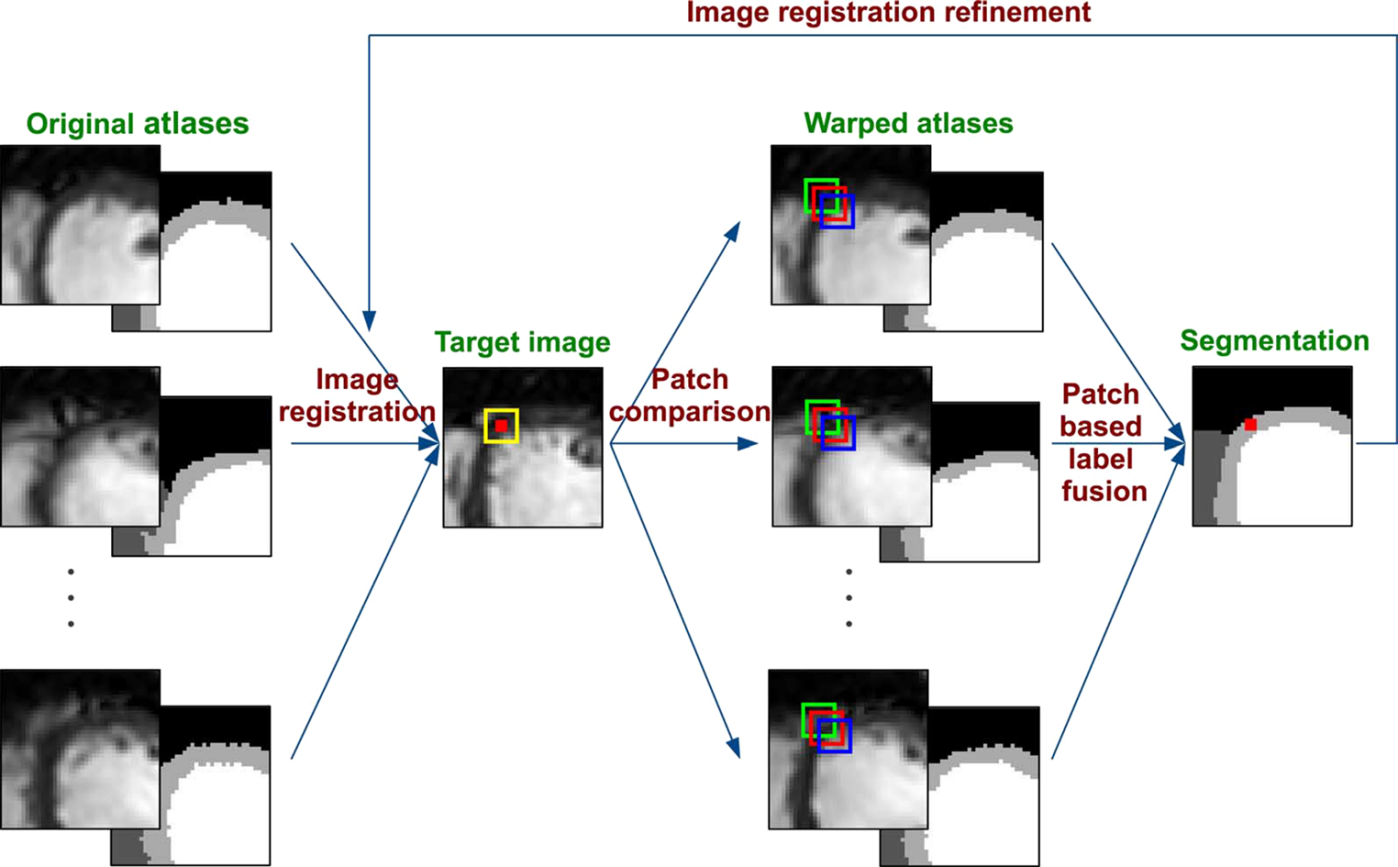
A framework of ventricular segmentation based on multi-atlas and label fusion technique. Atlases are first registered to the target image. The label at a voxel (*red dot*) is given by the comparisons between the patch (*yellow*) on the target image and the patches (*colourful boxes*) on the warped atlases, weighed by the distance and similarity. Then the fusion of labels from all atlases assigns each voxel a final class. The segmentation result is used to refine the registration process [156]

Valdes et al. [155] propose to use a probabilistic atlas to guide the EM classification. The atlas provides a spatially and temporally varying probabilistic map for the LV, RV, myocardium, and background including the liver, stomach, lungs, and skin. The results of estimated volume of LV, RV, and myocardium demonstrate that the combination of the EM algorithm and a cardiac atlas improves segmentation accuracy. Bai et al. [156] fuse patched-based labels for a Bayesian formulation within a multi-atlas registration based segmentation framework. Furthermore, they refine the registration using intermediate label information. Figure 13 illustrates the procedure of multi-atlas label fusion and image registration refinement.

Ordas et al. [157] introduce a feature vector, which is invariant under Euclidean transforms in an ASM-based framework. Mitchell et al. [158] propose a hybrid AAM matching mechanism accomplished through three steps. Firstly, AAM alone is fitted to the image. Next, the hybrid AAM/ASM helps avoiding local minima by deploying the shape information. Finally, AAM is reapplied. Zhang et al. [159] also use a combined AAM-ASM model, which is based on novel spatial and temporal features, incorporating the motion. The combination of ASM and AAM yields better segmentation results and overcomes the drawbacks of using ASM or AAM individually. ASM requires good initialisation and can be trapped by incorrect nearby features, though it retains a fine global shape. While AAM performs well in tracking objects, but is easy to be trapped by local minima. Alba et al. [160] segment the LV and RV of highly abnormal hearts by using estimating a mapping between the abnormal image and the space of generic shape model built from a normal population, which can be thus used to segment any types of cardiac abnormality. Increased accuracy is demonstrated for both pulmonary hypertension and hypertrophic hearts.

#### Direct estimation

**Fig. 14:**
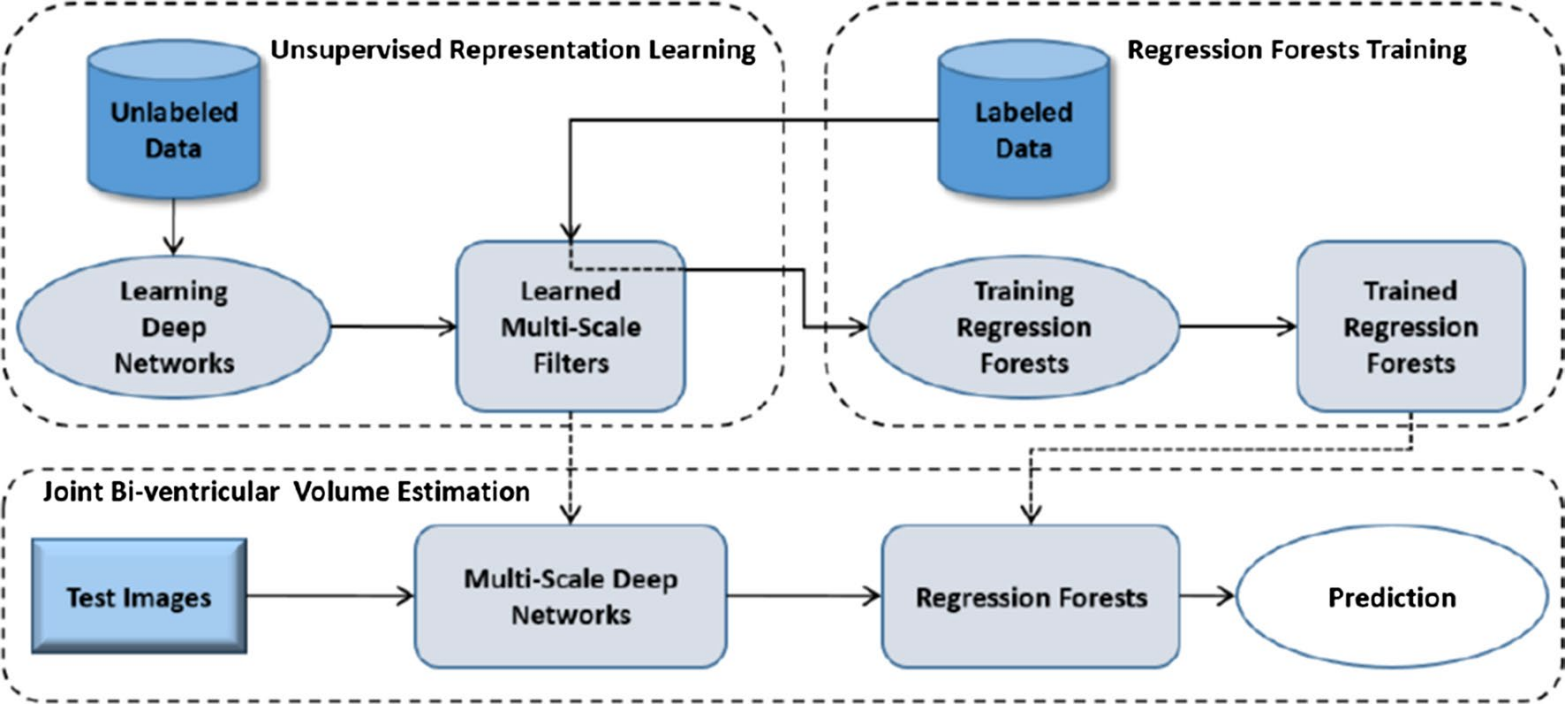
A framework of direct estimation: unsupervised learning searches an efficient image representation way and regression forest trained by using manually segmented data captures the discriminative features [16]

Wang et al. [15] estimate LV and RV cavity volumes on SAX slices without segmentation. This direct method relies on a likelihood function defined as the area correlation of the LV/RV cavities, and prior function specified by the product of the blobness, edgeness, and homogeneity. The framework consists of a training stage where the prior and likelihood probability functions are inferred. Given a test image, the posterior probability of observing a point in LV/RV is derived using the Bayes rule. The mean cavity area of LV/RV is the expectation of a function of these posterior probabilities and the volumes are estimated using Simpson’s method. However, as indicated by Zhen et al. [16], the limitations of [15] include a simple linear relationship assumption between LV and RV as well as an expensive computational requirement. Zhen et al. [16] make use of a three-layer convolutional deep network, which is learned from unlabelled images, to represent the input test case effectively in feature space. At the meantime, regression forests trained from manually labelled data, as discriminative learning, is responsible for estimating LV and RV volumes (see the flowchart in Fig. 14). They claim that their method significantly outperforms level-set and graph-cut methods. Another advantage of direct estimation is that the inconsistency of boundary and region intensity homogeneity has been excluded from the immediate influences on volumetric quantification.

### LA

Segmentation of the LA is more challenging compared to the other structures in the heart. The shapes of LA may have different variations and its blood pool consists of other structures such as the auricular appendage and pulmonary veins; the surrounding pulmonary artery and the aorta have similar intensities to the atrium in MRI and the LA is typically much smaller than the ventricles, showing a relatively thin myocardium. The activity of mitral valve also makes the boundary between LA and LV invisible under some cases [12]. As a consequence, computer-aided LA segmentation has obtained much less progress. We list the methods in Table 5.

#### Image-driven techniques

John and Rahn [161] base their approach on thresholding and region merging. A thresholding roughly separates the blood pool voxels from the image. Afterwards, the Voronoi tessellation of the binarised mask is computed. The tessellated components are finally combined to segment the LA and other structures. Zhu et al. [162] also propose a region-growing framework, where the initial seed is found according to the anatomical knowledge from the middle SAX slice. A shape prior learned from training data is used to attract the growth to the statistically plausible region. This incorporation makes segmentation more robust to spatial variation and image quality.

#### Model-driven techniques

**Fig. 15:**
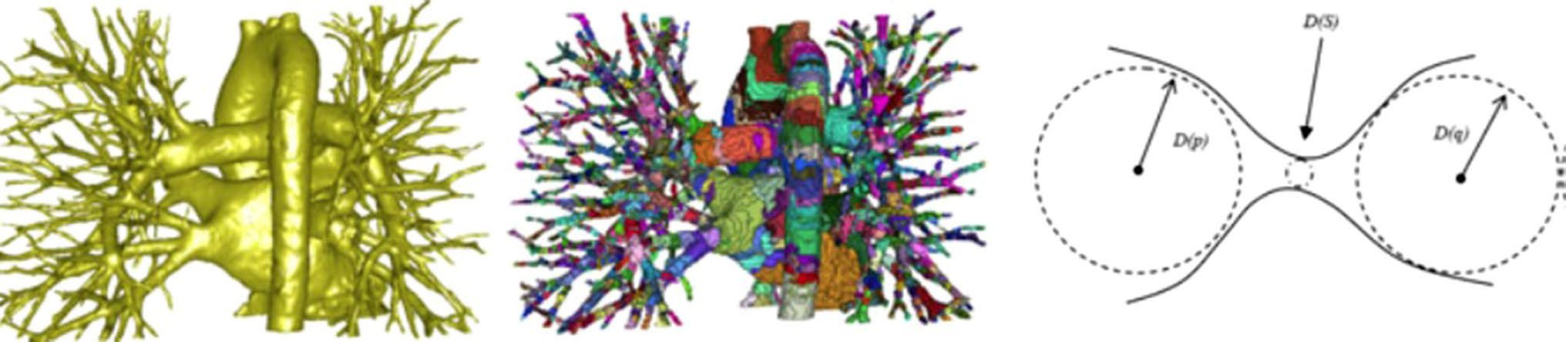
An LA blood pool (*left*) subdivided to Voronoi cells (*middle*). The narrow junction is the smaller sphere (*right*) locating between two larger components [163]

Karim et al. [163] construct a probabilistic atlas for atrium using 20 manually segmented training images. Given a test image, they apply an optimal thresholding to extract the blood pool and the vessel structures and obtain the Voronoi tessellation for the binarised image [161]. The narrow junctions, which are the connections between the atrium and its neighbouring structures, are then identified (as displayed in Fig. 15). Next, using the probabilistic atlas as prior, they present an MRF based cost function for segmenting cells that belong to the atrium. Additionally, a graph-cut method is applied for global optimisation. In order to deal with LA anatomical variations, Kutra et al. [164] have proposed a multi-component-based LA segmentation. The three most typical variations include the normal, common left trunk (CLT), and right middle pulmonary veins pattern (RMPV). Then a trained SVM is used to automatically select the model that fits the test image best. Eventually the model, which is a mesh of triangles, deforms towards the edge by the external and internal constraints.

### Whole heart

The objective of whole heart segmentation includes delineation of LV, RV, LA, RA, and great vessels if required. Because of tissue diversity and indistinct boundaries between substructures; however, limited works have proved good efficacy in whole heart segmentation. The methods discussed in our review are summarised in Table 6.

#### Image-driven techniques

Makowski et al. [165] have proposed an active contour-based procedure to segment the heart and vessels in 2D transversal slices. This shape-independent method uses a balloon force to place the segmenting contour roughly and then uses a snake model to refine the segmentation. However, due to the complex geometry of the whole heart, later published works tend to use shape priors to increase robustness.

#### Model-driven techniques

**Fig. 16:**
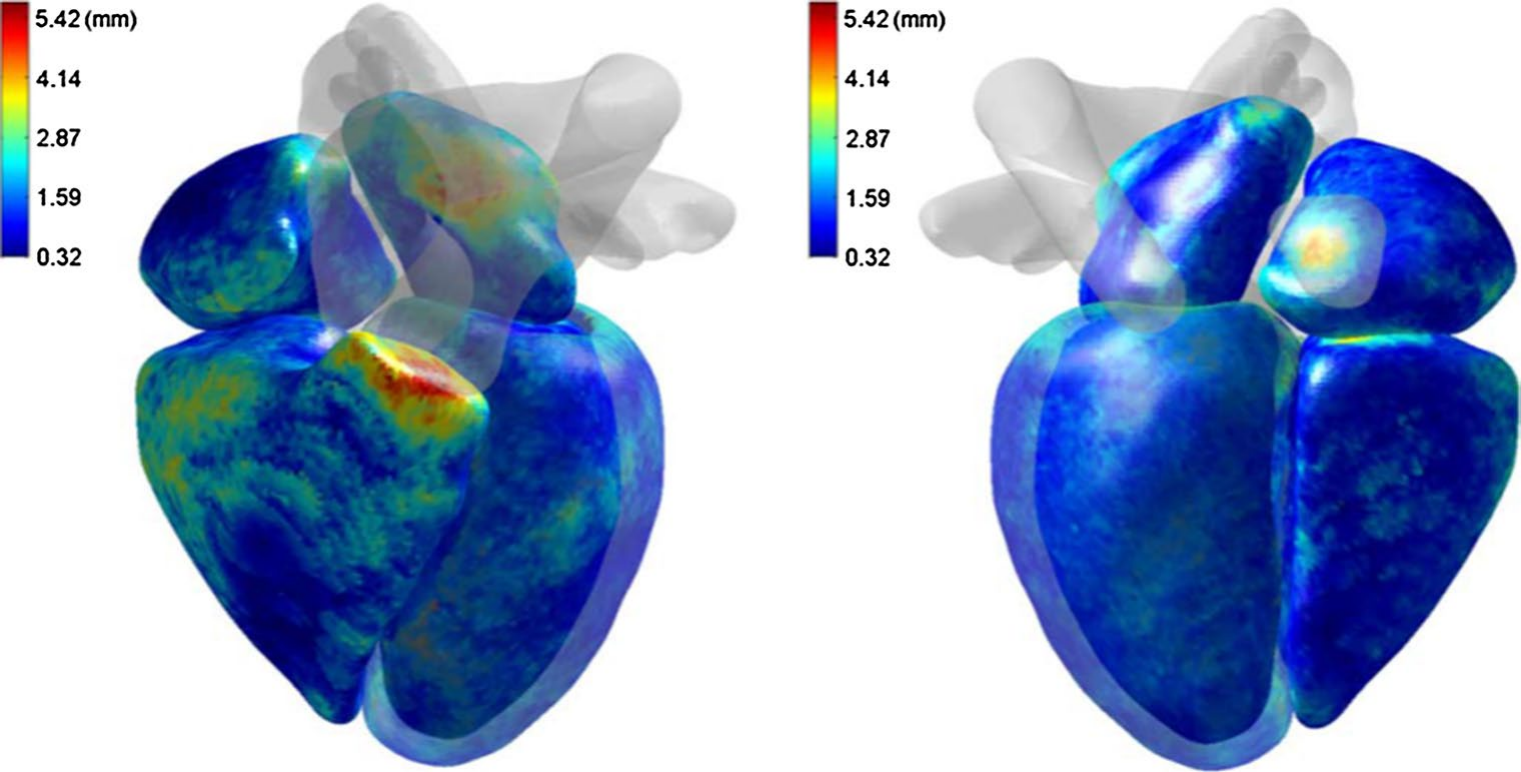
An evaluation of segmentation accuracy using surface-to-surface (S2S) distance between the segmented result and the manually delineated ground-truth from two different views in 3D [118]

Lotjonen et al. [166] reconstruct the 3D geometry of atria and ventricles from both SAX and LAX views. The pulmonary artery, pulmonary veins, and vena cava are excluded for volumetric measurement. The shape variability is modelled using PDM, a novel landmark distribution model, and a probabilistic atlas. Then the mean shape model is non-rigidly registered to the test image, and the model deforms towards the boundaries based on the shape priors. Since the performance of SSM-based methods is related to the richness of training samples, Koikkalainen et al. [167] have shown the feasibility to improve the segmentation of four-chamber and major vessels by artificially enlarging the training sets. Wierzbicki et al. [168] build PCA-based models for LV, RV plus RA, LA plus aorta, and the entire heart separately, using high quality training data. Each model is then registered to the mid-diastole frame of a low quality sequence, and propagated to all other frames by animating motion dynamics. Peters et al. [169] developed a deformable model by proposing a novel and robust boundary identifying technique called simulated search, whose mesh matching functions are previously trained. For the prior information-based approaches, model registration is always a critical step. Zhuang et al. [170] and Zuluaga et al. [171] find the breakthrough herein. They present a locally affine registration mechanism, which is further refined by a free-form deformation registration. This atlas-propagation-based method has turned out to be robust against various pathologies. Examples of the segmented whole heart in different views and a visualisation of segmentation errors between the result and the ground-truth are shown in Fig. 16.

#### Direct estimation

Zhen et al. [17] explored the feasibility in applying direct estimation to four-chamber volume measurement as well, by representing the MR images in a compact and discriminative way. The image features are generated using a supervised descriptor learning algorithm. Then the volume estimation becomes a multi-output regression problem solved with random forest.

## Discussion

Despite the advances in cardiac image segmentation listed in this review, there are plenty of challenges waiting to be addressed to allow a more comprehensive assessment of cardiac function in clinical practice and medical research with MRI.

### Choice of segmentation techniques

From this review, it can be seen there is a wide range of techniques and approaches that can be used for cardiac MR image segmentation. The choice of a particular technique is thus not trivial. However, a number of recommendations can be made. Firstly, the choice of the technique to be implemented can be constrained by the specific protocol. For example, a model-based technique can be used to obtain the walls of the LV is combined with thresholding to eliminate the effect of the papillary muscles. Secondly, the choice of a particular approach can depend on the availability of large training datasets. In such situations, model-based approaches can be very powerful tools to restrict the segmentation results to valid instances. In the contrary, when only small cohorts are available for training, model-based techniques can be too restrictive and methods that do not use any prior are preferred.

Finally, the obvious criterion for the choice of techniques should be the segmentation accuracy. However, while we provided a detailed list of the evaluations of the existing techniques in Tables 2, 3, 4, 5, and 6 for an overview of their performance, their direct comparison is difficult as the error metrics between the segmentation outcomes and the ground-truth are defined differently in different articles (point-to-surface errors, point-to-point errors, Hausdorff distance, dice similarity, correlation and linear regression coefficients, etc.). Also, the datasets are not the same in terms of image sequences, their numbers (sample size), and the classes (healthy vs. abnormal cases). For this reason, the emergence of challenges in international conferences is a very important initiative that will be able to highlight more objectively the merits and limitations of the existing methods. We can list, for example, the Left Ventricle Segmentation Challenge^1^ (*MICCAI 09*), Right Ventricle Segmentation Challenge^2^ (*MICCAI 12*), as well as Left Atrial Segmentation Challenge^3^ (*MICCAI 13*).

### Segmentation of the whole heart

**Fig. 17:**
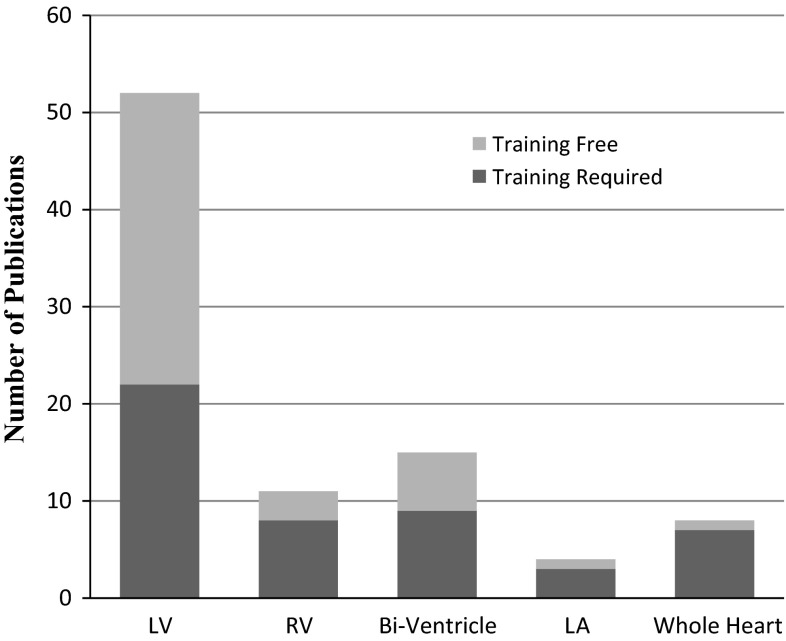
The amount of referred publications in each section

Among the four chambers, the LV has received the most attention in cardiac segmentation and MRI-based cardiac functional assessment. This is because it plays a key role on the process of the blood circulation, and thus its function/dysfunction is associated with most cardiac diseases. Furthermore, the LV has a relatively simple geometry with thick myocardial walls, making its automated segmentation more feasible. In contrast, as it can be seen through comparing the list of works reviewed in this paper (Tables 2, 3, 4, 5, 6), the RV and LA have received less attention from the cardiac image analysis community (Fig. 17). This is due to the more complex geometry of these chambers and their much thinner walls. Yet, these chambers are associated with many critical diseases, such as modelling in patients with pulmonary hypertension [160] or left atrial enlargement [57–59]. Further research is thus required to develop techniques capable of coping with the difficulties of segmenting complex and thin structures such as the RV and RA, and more generally to segment the whole heart to enable an assessment that takes into account the combined motion of all chambers.

### Segmentation of large-scale CMR datasets

Another future perspective is related to the segmentation of large-scale datasets. In the era of big data, there is a demand for computational techniques that are scalable for the processing of thousands of cases and for the extraction of novel clinical knowledge from existing databases. However, previous cardiac analysis methods in MRI have been developed and validated with at most a few dozen cases, often with well-controlled imaging protocols, and based on a homogeneous class of subjects (e.g. healthy). A major research topic in the future will consist of extending the existing techniques such that they can handle the large variability in anatomy and MRI image sequence that are typically found in large-scale databases. Furthermore, current cardiac MR segmentation methods are rarely fully automatic. User interaction often used for example to define manually the apical and valve points. However, this becomes impossible when dealing with large numbers of datasets, and thus fully automatic techniques will be required.

### Segmentation of abnormal cases

One major research challenge in cardiac segmentation is the development of approaches that are robust to different groups of individual and classes of disorders. In the existing literature, however, most techniques have been mainly developed and validated with normal subjects, and in some exceptional cases with mildly abnormal hearts, i.e. mostly few regional septal defects such as hypertrophic cardiomyopathy (HCM) [124, 135]. These techniques are developed in a generic form for both normal and abnormal cases and do not have a mechanism to handle explicitly large remodelling effects owing to cardiac diseases. Recently, Alba et al. [160] developed a technique specifically designed to segment severely abnormal hearts, with a promising validation to pulmonary hypertension patients with highly remodelled RV. Such techniques need to be further investigated using large cohorts and with multiple diseases to make the tools more robust for clinical use, where routine cardiac MRI quantification is concerned mostly with diseased subjects or subjects suspected to be diseased.

### Clinical translation

Finally, significant effort is being dedicated, in parallel to the consolidation of the cardiac image analysis techniques, to the clinical translations of software tools that can be used robustly and routinely in clinical practice. Table 7 presents some of the existing software used in clinical practice or in cardiovascular research in alphabetical order. We recommend the readers to check the details of the available software on their websites as the functionalities tend to evolve continuously over time as the result of new advances in CMR research.

**Table 7:** Examples of existing software platforms for cardiac structural and functional analysis with CMR

Name	Producer	Use	Website
CAAS MRV	Pie Medical Imaging	C	piemedicalimaging.com
CAIPI	Mevis Fraunhofer	R	mevis.fraunhofer.de
Corridor4DM	INVIA (Siemens)	C	inviasolutions.com
CMRtools	Cardiovascular Imaging Solutions	C/R	cmrtools.com
CVI42	Circle Cardiovascular Imaging	C	circlecvi.com
GIMIAS Cardio Suite	CISTIB	R	gimias.org
Heart IT	Heart Imaging Technologies	C	heartit.com
iNtuition Cardiac	TeraRecon	C	terarecon.com
PiA CMR	Precision Image Analysis	C	piamedical.com
Qmass	Medis	C	medis.nl
Segment CMR	Medviso	C	medviso.com
Ziostation MR Cardiac Function	Qi Imaging	C	qiimaging.com

*C* commercial, *R* research

## Conclusions

This review paper has summarised the most recent advances in cardiac image segmentation methods, which can be employed for the assessment of cardiac structure and function with CMR. These approaches range from image classification based techniques to statistical shape models. We have highlighted the properties of each of these approaches and their links to cardiac structure and functional assessment in MRI. After years of continuous developments, cardiac segmentation has become an interdisciplinary subject associating cardiology, medical imaging, and image processing. Further research is required to consolidate these advances with validation to larger cohorts, as well as to extend these approaches to the segmentation of all chambers and pathological hearts, ultimately allow for a more comprehensive application of the existing tools in clinical practice.

## Footnotes

1 http://smial.sri.utoronto.ca/LV_Challenge/Home.html.

2 http://www.ncbi.nlm.nih.gov/pubmed/25461337.

3 http://www.cardiacatlas.org/web/guest/la-segmentation-challenge.
